# Linking Measures of Inbreeding and Genetic Load to Demographic Histories Across Three Species of Bears

**DOI:** 10.1111/eva.70133

**Published:** 2025-07-16

**Authors:** Heather R. Clendenin, Matthew D. Pollard, Emily E. Puckett

**Affiliations:** ^1^ Department of Biological Sciences University of Memphis Memphis Tennessee USA

**Keywords:** conservation genomics, deleterious mutations, demographic history, inbreeding, runs of homozygosity

## Abstract

Historic and contemporary demography affect deleterious variation and inbreeding depression, meaning that measuring genetic diversity alone does not capture the nuances of genetic erosion. Contrasting genomic signatures generated by long‐term evolutionary processes to those generated by contemporary changes may help differentiate between populations more or less likely to persist with low diversity or high genetic load. To better understand these interactions, we examined signatures of inbreeding and genetic load across three species of bears: American black ( *Ursus americanus* ), brown ( *U. arctos* ), and polar ( *U. maritimus* ). We sampled across each species' geographic range to represent intraspecific variation in demographic history and ecology. We found that ROH burden often varied more among populations within lineages of species than between species. Admixed populations generally had higher heterozygosity and lower ROH burden; this pattern reversed in small, isolated populations. Greater diversity, including harmful variation, was found in larger, admixed populations—especially those with higher historical effective population sizes (N_E_). However, this did not necessarily correspond to more realized genetic load. While polar bears had low N_E_ and low realized load, brown and American black bears exhibited less realized load as N_E_ increased and greater realized load in populations with recent bottlenecks and/or indications of recent consanguineous matings. This vantage offers insight into genetic health and threats of genetic erosion within populations and species, which can meaningfully contribute to assessments of threat status. In American black bears, the composite of these metrics revealed a trend in the Louisiana population that may be diagnostic for management intervention based on contemporary demographic changes. In brown bears, the Apennine bear consistently fell outside of the range of values in other populations, reinforcing previous descriptions of isolation, inbreeding, and purging in this population. In polar bears, there were no regional trends that warranted concern with respect to genetic erosion.

## 1 Introduction

The relationships between genetic diversity, genetic load, and demographic history impact the likelihood of long‐term persistence of species. Populations of conservation interest are often assessed for overall genetic diversity as a measure of genetic health, which can be treated as a proxy for adaptive potential, i.e., the ability of populations to rely on standing genetic variation to provide fitness advantages under changes to selective pressures (Booy et al. 2000; Boulding 2008; Funk et al. 2019; Willoughby 2015). Adaptive potential is a particularly important aspect of persistence in the face of ongoing global change. Harmful or deleterious variation, i.e., genetic load, can also affect the fate of populations and is proposed to have a greater impact when population size is greatly reduced (i.e., bottlenecked) and inbreeding occurs (Mathur and DeWoody 2021). Generally, the proportion of harmful alleles within a population is correlated to the amount of neutral variation (Mathur, Mason, et al. 2023), but this proportion can be altered as a consequence of population demography and demographic history, e.g., increasing following precipitous bottlenecks or decreasing after prolonged low effective population size (N_E_) (Benazzo et al. 2017; Bertorelle et al. 2022; Kardos et al. 2017; Mathur and DeWoody 2021). Overall genetic diversity and the proportion of diversity comprising potentially harmful variation can affect the necessity and success of management interventions, such as genetic rescue (DeWoody et al. 2021; Kyriazis et al. 2021).

All individuals carry harmful genetic variation via deleterious alleles known as genetic load (Charlesworth et al. 1993). Inbreeding depression occurs when these deleterious alleles are frequently expressed because more individuals within a population are homozygous or when increased homozygosity leads to loss of heterozygous fitness advantage (Charlesworth and Charlesworth 1999; Frankham 2003; Hedrick and Garcia‐Dorado 2016). This can shift the proportion of genetic load that is considered “potential load” (i.e., harmful recessive variants masked in a heterozygous state) to “realized load” (i.e., harmful recessive variants in a homozygous state) (Mathur and DeWoody 2021). As such, populations with low observed heterozygosity and high estimated inbreeding are assumed to warrant management concern. An often‐used proxy measure of genetic health is the average heterozygosity of individuals within a population (Grueber et al. 2008). Additionally, Wright's inbreeding coefficient, *F*_IS_, calculates the relationship between the expected and observed number of heterozygotes within a population to estimate levels of inbreeding (Wright 1951).

As whole genome sequencing (WGS) becomes more tractable for non‐model organisms, we can gather indirect estimates of genetic load from genomic annotations and quantify runs of homozygosity (ROHs) across genomes to measure inbreeding (Fitzpatrick and Funk 2021; Kardos et al. 2016). ROHs are stretches of homozygous regions that are identical by descent, meaning they originated from a shared ancestor (Ceballos et al. 2018). They indicate consanguineous mating (i.e., mating between relatives) has taken place among the individual's ancestors, and the proportion of the genome comprised of ROHs (*F*_ROH_) can serve as a genomic inbreeding coefficient. Any recessive deleterious variants present within ROHs are expressed, which can have weighty consequences when consanguineous matings occur. ROHs tend to be enriched for deleterious variants relative to other genomic regions (Szpiech et al. 2013). Given that certain types of mating events are more common under particular demographic scenarios (e.g., inbreeding following population bottlenecks; matings between highly outbred individuals following an admixture event), patterns in ROH tract length and frequency are affected by both long‐ and short‐term demographic history (Ceballos et al. 2018; Curik et al. 2014; Kirin et al. 2010). The lengths of ROH tracts tend to be greatest immediately following a consanguineous mating event and decrease with subsequent generations due to recombination (Kirin et al. 2010). Longer, more recently formed ROH tracts harbor more deleterious variants than shorter, older ROH tracts, where purifying selection has had more time to purge harmful variants (Stoffel et al. 2021; Szpiech et al. 2013). As such, scrutiny of ROHs may be particularly fruitful when interested in inbreeding depression.

Demographic history shapes patterns of genetic diversity and load among populations, which subsequently alter the consequences of inbreeding on genetic health. Populations with larger historic effective population sizes (N_E_) generate greater genetic variation. However, overall amounts of genetic diversity are correlated with amounts of harmful variation. High diversity may become a liability when large populations experience reductions, which increases the likelihood of consanguinity and shifts potential load to realized load (Kyriazis et al. 2021; Mathur and DeWoody 2021). Population size also determines whether selection or drift plays a greater role in changes in allele frequencies. Smaller historic N_E_ has been associated with a proportionally larger burden of harmful alleles, suggesting that selection is more effective at removing deleterious variation in larger populations (Wilder et al. 2023). Prolonged bottlenecks can also fix deleterious variants as realized load (Bertorelle et al. 2022). As a counterpoint, genetic purging—i.e., the removal of harmful alleles, typically highly deleterious, when exposed to selection in homozygous states—is expected to occur in populations with low N_E_ and stable selective pressures over a sufficiently long period of time. Populations that have purged genetic load do not experience significant fitness reductions when inbreeding takes place because it does not significantly increase the homozygosity of deleterious variants (Bertorelle et al. 2022). Successful captive breeding programs have demonstrated targeted reduction in variants underlying inbreeding depression during demographic rescue of small, necessarily inbred populations (Templeton 2002; Templeton and Read 1984). However, this process is not to be conflated with populations that already have reduced fitness caused by very low genetic variation, wherein inbreeding may not lower fitness further.

At present, there are no explicit metrics pertaining to genetic health included in IUCN threat status criteria. Genetic diversity targets, such as the metric described by Jeon et al. (2024), have been proposed for inclusion among overall efforts to halt biodiversity loss by the Convention on Biological Diversity (CBD), particularly within the Kunming–Montreal Global Biodiversity Framework (GBF) (Hoban et al. 2021, 2024). Due to the limitations and urgency of conserving global biodiversity, genetic indicators of extinction or extirpation risk must be tractable for use by managers and policymakers while remaining scientifically defensible (Hausdorf 2021; Hoban et al. 2024). Resources vary widely across taxa and geographic regions, tending to skew towards charismatic megafauna and regions of higher economic development (dos Santos et al. 2020; Troudet et al. 2017). Consequently, IUCN threat status is not a reliable indicator of genetic diversity or genetic load (Bertorelle et al. 2022; Brüniche‐Olsen et al. 2018; Schmidt et al. 2023). To allow for a broader and more inclusive scope of assessments, less resource‐demanding indicators, such as census population size estimates for data‐poor species and regions, have been recommended as proxies for direct estimates of genetic diversity (Hoban et al. 2024).

In more data‐rich study systems, interest is increasing in characterizing the various relationships between genetic diversity, genetic load, and demographic history across wild organisms for the sake of conservation and management (DeWoody et al. 2021; Fitzpatrick and Funk 2021; Grossen et al. 2020; Xue et al. 2015). Current studies have varied in their sample selection and the taxonomic and geographic range they represent. For instance, within a single species, some studies have focused on single populations (Humble et al. 2023; Robinson et al. 2019), while others contrasted the demographic trends in multiple subspecies and/or populations of the same species (Armstrong et al. 2021; Mathur, Tomeček, et al. 2023). In multi‐species studies, contrasts have been made between closely related species representing different demographic histories (Wootton et al. 2023) and single individual studies with broad taxonomic sampling (Brüniche‐Olsen et al. 2018). Additionally, research in non‐model organisms tends to skew towards endangered species and populations, with less data on healthy populations and non‐threatened species, making it difficult to determine a “baseline” for these metrics (Bosse and van Loon 2022).

We have chosen to look at genomic indicators of genetic health and demographic history within a comparative and hierarchical framework, i.e., sampling populations and individuals across the ranges of three closely related bear species: American black bear ( *Ursus americanus* ), brown bear ( *U. arctos* ), and polar bear ( *U. maritimus* ). Together with other large‐bodied organisms, bears are at higher risk of local extirpation, and their loss has an outsized effect on their ecological communities (Doyle et al. 2015; Séguin et al. 2014). Conservation strategies targeting bears allow them to act as umbrella species, providing benefits to other species that share their large habitats. Additionally, bears share many resources with humans, so their persistence on landscapes entails carefully balancing conflict management and habitat protections (Penteriani and Melletti 2020). These species are well‐studied and tend to be data‐rich, including in genomic data, relative to many other non‐model organisms. There is also variation in the conservation status both between these species and among their populations. Given that there are not currently assessments included in IUCN to monitor the threat of genetic erosion, which is a risk of fragmented, small, and declining populations, deeper considerations of the genetic health of these species are warranted (Schmidt et al. 2023).

American black bears continue to admix following expansion from glacial refugia, with recent fragmentation and isolation primarily in the southern portion of their range (Murphy et al. 2018; Puckett et al. 2015; Puckett and Davis 2021). Black bears are split between three lineages: western, eastern, and a genetic cluster in Alaska characterized by isolation‐by‐distance from the eastern lineage and admixture with the western lineage (Puckett et al. 2015). As a species, American black bears are increasing in number, and their global population is estimated to be more than twice that of all other bear species combined (Garshelis et al. 2016). American black bears are generalists and behavioral opportunists, broadly distributed across North America and primarily adapted to temperate and boreal forests (Penteriani and Melletti 2020). However, they also are found in Mexican scrub forests and deserts, Labrador tundra, Pacific coast rainforests, and Louisiana swamps, and are known for their ability to acclimate to human‐altered landscapes. Habitat loss and fragmentation currently pose the largest threat to a few populations, where the consequences of small population size and isolation are exacerbated by heightened levels of human‐bear conflict (Garshelis et al. 2016).

Brown bears have the most extensive range out of all bears and persisted in small but connected populations until humans transitioned to agriculture. This transition resulted in populations in southern Europe becoming mostly small and isolated, while populations in northern Europe/Fennoscandia remained large and interconnected (Benazzo et al. 2017; Kopatz et al. 2014). Brown bears fall into three to eight lineages depending on the method of assignment but can be broadly divided into four geographic groups associated with the Gobi Desert and the Himalayas, Europe, eastern Asia, and North America (de Jong et al. 2023; Tumendemberel et al. 2023). They occupy a more diverse suite of habitats than any other bear species, including temperate rain forests, dry steppes, and Arctic shrublands, and exhibit local adaptations suited to these habitats (Tumendemberel et al. 2023). Conservation status varies among different populations: some populations are considered stable, in recovery, or facing ongoing threats due to the loss and fragmentation of habitat, pollution, and human‐bear conflicts (Huber 2025).

Polar bears are distributed across four polar ecoregions (i.e., polar basin convergent, polar basin divergent, archipelago, and seasonal ice), but genetic data indicate that they may be best characterized as a single meta‐population with a long history of low N_E_ (Amstrup et al. 2008; Laidre et al. 2022). They are highly adapted to polar sea ice environments; this specialization, combined with their demographic history, is likely responsible for genetic purging across the species (Liu et al. 2014). However, the polar bear conservation status is “vulnerable,” according to the IUCN Red List of Threatened Species (Wiig et al. 2015). This is primarily driven by the loss of sea ice habitat due to climate change, which poses a severe threat to this highly specialized species (Atwood et al. 2016). Additionally, oil and gas development in the Arctic, shipping activities, and potential increased human‐bear interactions due to changing ice conditions create additional conservation concerns (Atwood et al. 2017).

In this study, we explore signatures of inbreeding and genetic load in terms of both contemporary bottlenecks and deeper demographic processes across three closely related bears. To understand these relationships clearly, we have sampled across the hierarchical structure within each species to compare among species and among populations. We used WGS to compare patterns in ROH tract distribution and measure genetic load. Given the challenges of procuring direct fitness data at this scale, we annotated variants to compare the distribution of putatively harmful alleles across the genome with respect to both potential and realized load. We also examined the relationships between ROHs and load within the context of ancestral population sizes based on coalescent modeling. Populations of American black bears in Louisiana and brown bears in the Apennines of Italy, which have pre‐existing scholarship on their conservation concerns, will be showcased and predefined as bottlenecked (Gervasi and Ciucci 2018; Murphy et al. 2018). We expected the different demographic histories, environmental selection pressures, and threat statuses of these bears to correlate with the patterns of ROH tracts and deleterious variation between groups.

## 2 Materials and Methods

### 2.1 Sample Collection

**FIGURE 1:**
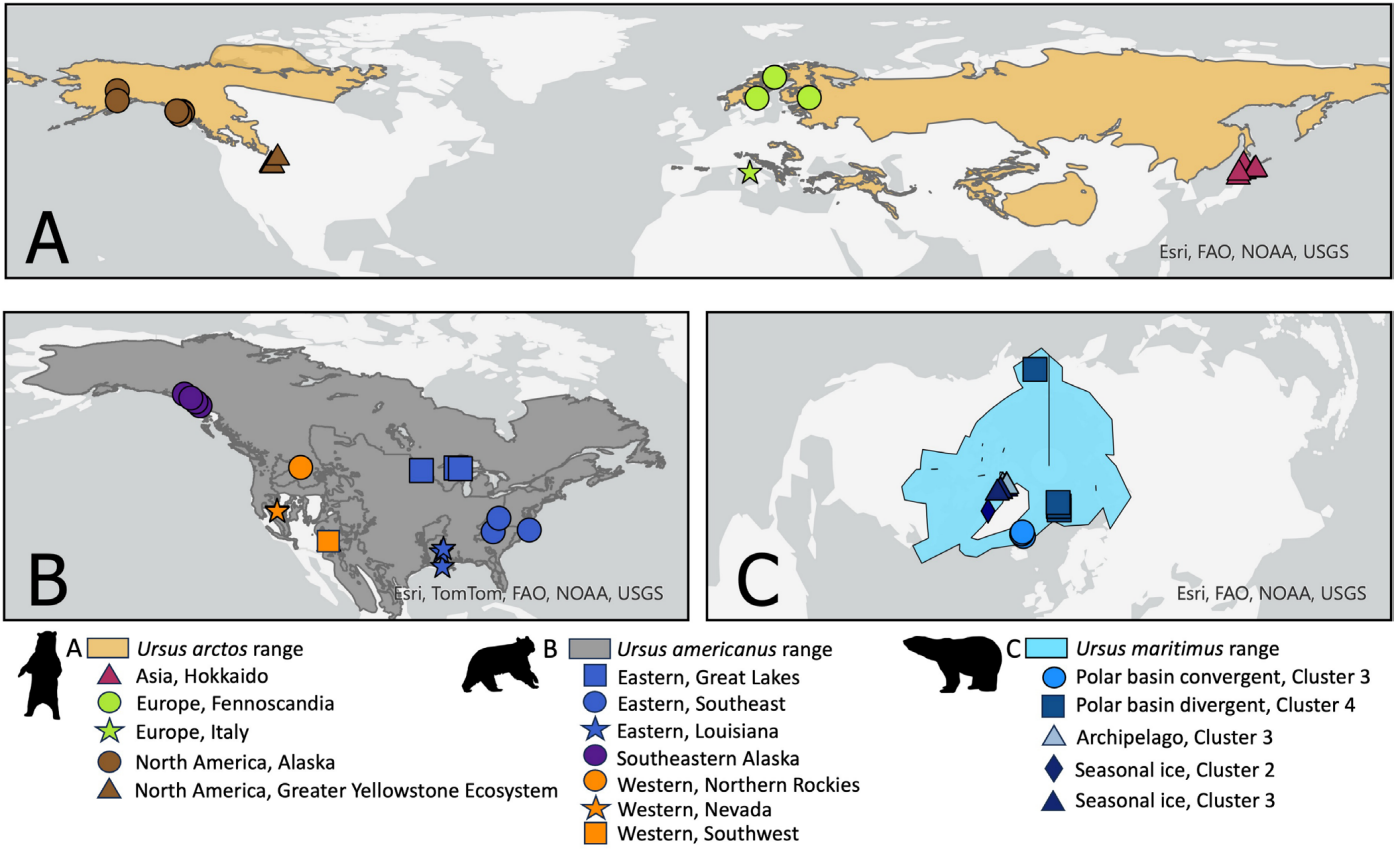
Range maps and sampling locations for each species based on IUCN data. (A) The range of brown bears ( *Ursus arctos* ) is depicted in light brown with sampling across the Asian (Hokkaido, Japan; maroon), European (bright green), and North American (dark brown) lineages. Within the European lineage, symbols represent animals from different populations, including Fennoscandia (circles) and Italy (Apennine; star/asterisk). Within North American brown bear samples from Alaska and the Greater Yellowstone Ecosystem are distinguished as circles and triangles, respectively. (B) The range of American black bears ( *U. americanus* ) is depicted in gray. Bears from the Eastern lineage are in royal blue, with symbols to denote three populations: Great Lakes (squares), Southeast (circles), and Louisiana (star). Western lineage black bears are depicted in orange, with populations spanning the Northern Rockies (circles) and Southwest (triangles) and Nevada (stars). Note that three individuals were sampled in Nevada and cannot be distinguished on this map. Samples from Southeast Alaska are represented by purple circles. (C) The range of polar bears ( *U. maritimus* ) is depicted in light blue, and individuals sampled from the polar basin convergent ecoregion are in dodger blue (and represented by filled circles corresponding to genetic cluster 3), individuals from the polar basin divergent ecoregion are in dark teal (and represented by filled squares corresponding to cluster 4), individuals from the archipelago ecoregion are in sky blue (and represented by filled triangles corresponding to cluster 3), and individuals from the seasonal ice ecoregion are in navy blue circles (represented by either filled diamonds for cluster 2 or filled triangles for cluster 3).

We downloaded WGS data for 19 black, 25 brown, and 25 polar bears. Samples of each species cover the geographic range (Figure 1) and specifically span previously described intraspecific variation. All samples were downloaded from the NCBI SRA, and accession numbers are listed in Table S1. Latitude and longitude were either obtained from direct GPS measurements, published data, or the nearest coordinates to descriptive locations.

### 2.2 Genome Processing

We mapped reads to reference genomes using the BWA‐mem algorithm in the Burrows–Wheeler Alignment Tool (BWA) package (Li and Durbin 2010). Reads for brown and polar bears were mapped to UrsMar_1.0 (GCF_000687225.1), which has a corresponding database in SnpEff and has scaffolds of sufficient length to detect ROHs (Liu et al. 2014). For American black bears, reads were mapped to two different reference assemblies due to downstream analysis needs. We mapped reads to ASM334442v1 (GCA_003344425.1) for SnpEff annotation because this database came pre‐loaded with annotations for this assembly (Srivastava et al. 2019). For the assessment of ROH, reads were mapped to ASM334442v1_HiC from the DNA Zoo initiative. This version used ASM334442v1 (GCA_003344425.1) as a draft assembly to construct chromosome‐length scaffolds (Dudchenko et al. 2017, 2018); we selected this assembly with longer scaffold lengths to facilitate more accurate estimates of longer ROH tracks. SAMtools v1.9 (Li et al. 2009) was used to estimate depth of coverage for samples, which ranged between 14 and 54.8× (American black bears: mean = 30.3, SD = 6.3; brown bears: mean = 28.4, SD = 13.9; polar bears: mean = 26.4, SD = 8.2; Table S1). Following mapping, duplicates were marked, and read groups were added or replaced using GATK v4.1.8 (McKenna et al. 2010) before indexing with SAMtools. We called variants using GATK then filtered each gVCF based on basepair (bp) quality score (GQ > 20) and genotype read depth (4 < DP < 300) before removing multiallelic SNPs and indels.

We used 36 well‐assembled autosomes (i.e., scaffolds 2–37) in the ROH analyses of American black bears, representing 89% of the total genomic length, with the shortest scaffold spanning 85.7 Mb. For brown and polar bears, there was not a similar demarcation in scaffold quality in the assembly; therefore, 238 scaffolds were selected, representing 96% of the total assembly, with the shortest scaffold retained spanning 1.05 Mb. PLINK v1.9 (Purcell et al. 2007) was used to remove shorter scaffolds. We verified that no scaffolds included in any analyses were associated with either the X or Y chromosomes (Bidon et al. 2015; Cahill et al. 2013).

### 2.3 PCA to Define Genetic Clusters

To confirm genetic clusters identified in the literature based on our sample selection, we ran a PCA in PLINK for each species. Using VCFtools v0.1.17 (Danecek et al. 2011), we thinned each species dataset to one site every 20 kb and to variants with a MAF > 0.05.

### 2.4 ROH Analyses and Assessment of Genetic Diversity

We detected ROH tracts using the sliding‐window‐based method in PLINK. We selected parameters to keep the scanning window small in order to detect short ROHs and avoid underestimating F_ROH_. These parameters included homozyg‐window‐threshold 0.05, homozyg‐window‐missing 5, homozyg‐window‐het 5, homozyg‐density 50, and homozyg‐het 1. We set the maximal gap to 500 kb (homozyg‐gap 500). Each scanning window had a minimum size of 100 kb (homozyg‐kb). The minimum number of SNPs per window (homozyg‐window‐snp) and the final ROH segment (homozyg‐snp) were based on species‐specific estimates of the number of SNPs per ROH intended to eliminate artificially small ROH due to chance (Gorssen et al. 2020; Purfield et al. 2012). These estimates were generated using the formula for the L‐parameter proposed by Lencz et al. (2007) and modified by Purfield et al. (2012) and account for the number of individuals genotyped, the number of genotyped SNPs, the percentage of false positive ROH, and the mean heterozygosity across all SNPs (Lencz et al. 2007; Meyermans et al. 2020). The L‐parameter estimates were 63 for *U. americanus* , 41 for *U. arctos* , and 61 for *U. maritimus* . To check the impact of this parameter, we tested each value (i.e., 63, 41, and 61) for each species and found that individual F_ROH_ varied by < 1% at most. We did not prune by linkage disequilibrium nor minor allele frequency, as this can result in missing long ROHs (Meyermans et al. 2020).

We measured individual‐level genetic diversity and generated ROH summary statistics for all sampled individuals. We estimated observed heterozygosity (H_O_) in two ways (Schmidt et al. 2021). First, we estimated “SNP heterozygosity” by counting the proportion of heterozygous sites from the total variable sites within each species using VCFtools. Second, we estimated “genome‐wide heterozygosity” as the proportion of heterozygous sites compared to the total number of sites mapped in the genome. We used the package detectRUNs (Biscarini et al. 2018) in R v4.1.2 (R Core Team 2021) to generate summary statistics from the PLINK ROH output files. These metrics included the proportion of the genome in ROH (F_ROH_), the number of ROH tracts within a genome, and the sum of ROH tract lengths in base pairs by individual. To illustrate differences in species‐specific patterns of ROH length distributions, custom Unix scripts (https://github.com/hclendenin/ROH_Load_Demography_Ursus) were created to categorize ROHs into three length classes: Short (100–400 kb), Medium (400–800 kb), and Long (> 800 kb).

### 2.5 Variant Annotation

SnpEff predicts deleterious variants within genic regions based on a combination of the sequence annotation (e.g., intron, exon, UTR, etc.) and the type of variant (Cingolani et al. 2012). To limit deleterious annotations to derived alleles only, we identified the ancestral *Ursus* alleles using the method described by Stapley (2020). Specifically, we mapped one sloth bear (*U. ursinus*) sample (ERR946786 from Kumar et al. 2017) to each of the reference genomes (see above). This sample was processed using the pipeline described above for our target species. Variants in this sample were treated as ancestral alleles and used to annotate the VCFs of our black, brown, and polar bears. Based on these annotations, we were able to determine whether the reference alleles at called sites in our target species were ancestral or derived, and then retained only derived allele calls for downstream analyses.

SnpEff annotations include the functional category of variants (e.g., synonymous, missense, splice region, etc.) and their predicted effect, specifically if the impact on gene function is low, moderate, or high/loss of function (the categories of these variants can be found in Table S1). Following annotation, we filtered variants by individual to allow comparison of deleterious load between populations and species. For each individual, we determined if deleterious variants were present in either the heterozygous (i.e., potential load) or homozygous (i.e., realized load) state via filtering with BCFtools view ‐q 0.05 and 0.55, respectively (Danecek et al. 2021).

### 2.6 Evaluation of Deleterious Variation across Genomes

To identify deleterious variants located within ROHs, we created separate BED files using the genomic coordinates of the annotated variants and the detected ROHs, then intersected these files using BEDTools v.2.31 (Quinlan and Hall 2010). Because we used different reference genomes to analyze American black bears, we first aligned the assemblies using Progressive Cactus v.2.5.1 (Armstrong et al. 2020). NCBI annotations were lifted over to the DNA Zoo assembly. Variants that aligned to more than one place in the DNA Zoo assembly (due either to small scaffolds or true synteny between genomic regions) were addressed by removing duplicate alignments and limiting analyses to the chromosome‐scale scaffolds. Of the 126,519 SnpEff‐annotated variants in the NCBI assembly, 125,979 remained following the lift over and removal of duplicate alignments, and 117,450 remained after subsetting to scaffolds 2–37. We then intersected the corresponding sets of BED files. Custom Unix scripts were used to generate counts of variants within ROHs and by ROH size (https://github.com/hclendenin/ROH_Load_Demography_Ursus).

### 2.7 Evaluation of Fixed Deleterious Variation within Species

The above approach identifies polymorphic variants within species but does not identify fixed variants that will be present in the reference genome. Thus, to estimate fixed variants, we remapped species to the other reference genome. Specifically, American black bears were mapped to the polar bear reference, whereas brown and polar bears were mapped to the American black bear reference. We chose this approach instead of mapping all species to a single outgroup to avoid overestimating fixed variants that were shared along the branch leading to these three species. Our approach aims to identify the uniquely fixed variants within each species.

We followed the mapping procedures outlined above, except for a change to the reference genome. Following merging samples into a single gVCF file, we filtered for derived variants with a frequency greater than 0.95. We then took this set of variants through the SnpEff annotation pipeline described above.

### 2.8 Historical Effective Population Size Estimation

We calculated the geometric mean of long‐term effective population size (N_E_) by estimating PSMC’ within MSMC2 (Wang et al. 2020). For this analysis, we selected a geographically representative subsample of bears from within species lineages due to computational costs. Using the BAM files produced following mapping to the respective bear reference genome, we reprocessed the allele calling as recommended by the authors by first building a pileup with the BCFtools mpileup, then calling sites with the call function, all within the provided bamCaller.py script. For each sample, we adjusted the depth parameter based on our estimated genome‐wide average depth calculated with the SAMtools depth function (Table S1). The recalling allowed positive mask files of sufficient depth for allele calling to be generated jointly with a vcf‐containing singletons. We did not phase haplotypes within species prior to making input files with the generate_multihetsep.py script. MSMC2 was run with the default time segment pattern 1*2 + 25*1 + 1*2 + 1*3 (Schiffels and Durbin 2014). To distill the change in N_E_ over time to a single point estimate, we calculated the geometric mean of lambda (λ) but excluded the first two and last three time segments (i.e., 1*2 and 1*3) as they are less accurately estimated, and we wanted to remove this uncertainty. We converted this estimate to N_E_ by taking (1/λ)/2 μ, using a mutation rate (μ) of 10^−8^ for all three species (Kumar and Subramanian 2002). Given the order of magnitude difference among species, all plotting utilized the log10 of the geometric mean (Wilder et al. 2023).

## 3 Results

### 3.1 Genetic Clustering

PCAs for American black and brown bears showed similar results for geographically structured lineage divergence across the range (Figure S1A,B) when compared to more comprehensive work (Puckett et al. 2015; Endo et al. 2021; de Jong et al. 2023). The PCA for polar bears (Figure S1C) indicated differences from the most comprehensive assessment of structure for that species (Laidre et al. 2022). This was largely due to sample selection that excluded the recently identified southern Greenland population, which has a large effect on the PCA due to isolation. Similarly to Laidre et al. (2022), the ecoregions Archipelago and Polar Basin converged, as defined by Amstrup et al. (2008), represent a single genetic cluster.

### 3.2 ROH Analyses and Assessment of Genetic Diversity

#### 3.2.1 American Black Bears

**FIGURE 2:**
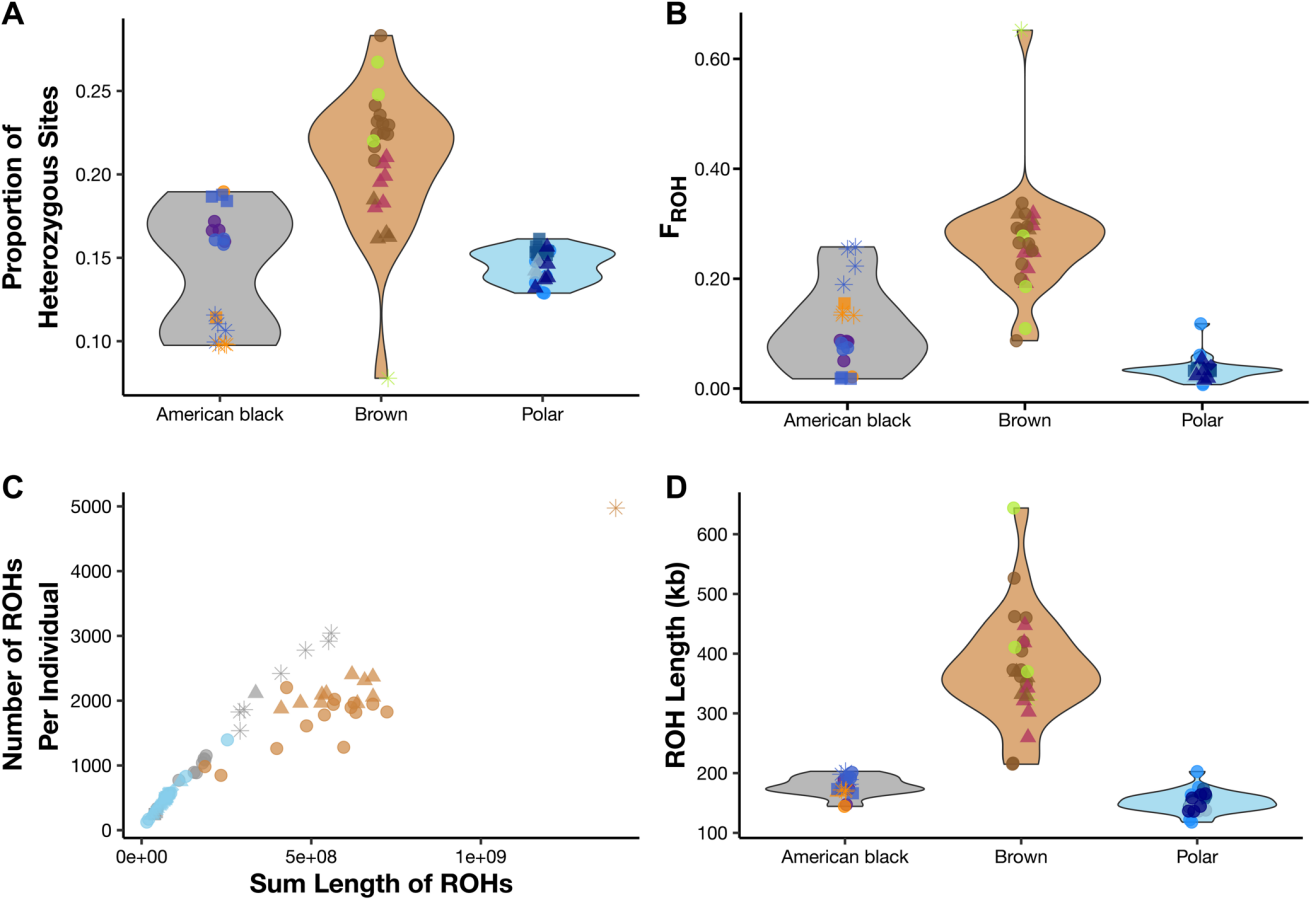
Comparison of diversity metrics among three bear species ( *Ursus americanus* : Gray; *U. arctos* : Light brown; *U. maritimus* in light blue; point symbology corresponds to samples from Figure 1). Individual estimates of (A) observed heterozygosity across all variable sites (i.e., SNP H_O_), (B) the proportion of the genome comprised of ROH tracts (F_ROH_), (C) the total sum of ROH tracts (in kb) relative to the number of ROH tracts across an individual's genome, and (D) the average length of ROH tracts by individual.

ROH analyses and measures of genetic diversity among American black bears varied more by population than by lineage. Individuals exhibited relatively moderate H_O_ and ranged from low to moderate levels of F_ROH_, where bears from small populations known to have been through a recent bottleneck, recolonizing following local extirpation, or naturally fragmented (Louisiana, Nevada, and Arizona, respectively; Figure 2A,B) (Gould et al. 2022; Malaney et al. 2018; Murphy et al. 2018) have higher proportions of the genome in ROH. SNP H_O_ was highest among individuals from the Great Lakes and Northern Rockies populations (H_O_ = 0.184–0.190; Figure 2A; Figure S1, Table S1), whereas individuals in Louisiana and the Southwest had the lowest (0.098–0.114). Values for genome‐wide H_O_ overlapped more across different regions but were generally higher among the Great Lakes, Northern Rockies, and Southeast Alaska (0.00140–0.00141) than among the Southwest and Louisiana (0.00139–0.00140; Figure S1, Table S1). F_ROH_ values followed an oppositional pattern where populations with lower heterozygosity had higher proportions of the genome in ROH tracks. The highest values occurred among Louisiana individuals (0.223–0.258), followed by individuals from the Southwest (0.133–0.155), Southeast Alaska (0.051–0.088), the Southeast (0.071–0.083), and the Northern Rockies (0.021), with individuals from the Great Lakes having the lowest F_ROH_ (0.018–0.021).

The greatest ROH burden, in terms of the number of ROHs, the sum of ROH lengths, and the average lengths of ROHs within individuals, was observed among individuals from Louisiana and the Southwest (Figure 2C,D). Individuals from Louisiana had the highest number of ROHs (2419–3043) and the longest total ROH lengths (411–559 Mb), followed by individuals in the Southwest (i.e., Nevada and Arizona: 1535–2115 ROHs, 245–336 Mb). Individuals from the Southeast and Southeast Alaska had intermediate values (771–1149 ROHs, 110–190 Mb), and the lowest number of ROHs and total tract length was found in the individuals sampled from the Northern Rockies (335 ROHs, 46 Mb) and Great Lakes populations (245–278 ROHs, 385–446 Mb). American black bears primarily carried few and short ROH tracts apart from the previously mentioned small, isolated populations. Per the ROH size classes (Figure S3), 97.74% of American black bear ROHs fell within the “short” (100–400 kb) class (25,930 ROHs), 2.21% (587 ROHs) fell in the “medium” length class (400–800 kb), and only 0.05% (13 ROHs) were greater than 800 kb.

#### 3.2.2 Brown Bears

Genetic diversity and ROH values among the brown bear populations and lineages generally overlapped, with the exception of a single outlier representing a unique and isolated population in the Apennine region of Italy (Figure 2A–C). SNP H_O_ of bears in Hokkaido (Asian lineage) ranged between 0.180 and 0.210. Most North American brown bears had SNP H_O_ values that fell in a similar range (0.161–0.283), with values generally lower for animals sampled in the Greater Yellowstone Ecosystem (SNP H_O_ < 0.184, Figure 2A). European brown bears were similar (0.219 < SNP H_O_ < 0.267), except for the bear sampled from Italy (SNP H_O_ = 0.078). This pattern was retained when looking at genome‐wide H_O_: The Apennine bear had the lowest value (0.0004), followed by the bears in the Greater Yellowstone Ecosystem (0.0007–0.009) and the Hokkaido bears (0.0009–0.0010; Figure S1, Table S1). Brown bears in Alaska and Fennoscandia had overlapping values of genome‐wide H_O_ (Alaska: 0.0010–0.0014; Fennoscandia: 0.0011–0.0013). F_ROH_ values were also similar across the range, falling between 0.087 and 0.337; however, the outlier Apennine individual had a value of 0.652 (Figure 2B).

Overall ROH burden showed a similar pattern as the distribution of F_ROH_ (Figure 2C,D). The total number of ROHs per individual fell between 847 and 2581 and the total length of ROH tracts fell between 234 and 560 Mb, with the same outlier as H_O_ and F_ROH_ (i.e., Apennine: ROHs: 4974; total length of ROHs: 1398 Mb). Relative to black bears, brown bear individuals had high numbers of ROHs in each length class: 2841 were short ROHs (82.28%), 6442 were classified as medium‐length (11.29%), and 46,483 fell in the long size class (5.43%) (Figure S3). Aside from the Apennine sample, there were no regional or lineage‐specific patterns in the total number of ROHs, total length of ROH tracts, or distribution of ROH classes across brown bears.

#### 3.2.3 Polar Bears

Relative to the other bear species, polar bear individuals exhibited low F_ROH_ (0.007–0.118, Figure 2B). SNP H_O_ was moderately low (0.099–0.161; Figure 2A), while genome‐wide H_O_ was very low (0.0002–0.0003; Figure S1, Table S1). While values across ecoregions and genetic clusters were largely intermixed, they were generally higher for Polar Basin divergent individuals assigned to genetic cluster 4 for both measures of H_O_. Given that heterozygosity and F_ROH_ are often thought to be complementary, it was notable that the individual with the highest F_ROH_ did not have especially low H_O_ (using either measure) relative to other polar bears.

Polar bear individuals generally exhibited a low ROH burden. They had a low total number of ROHs (123–1394) and total ROH lengths (15–253 Mb; Figure 2C). Among these few ROHs, 99.48% were short (17,503 ROHs), 0.52% were medium‐length (136 ROHs), and only two bears had any long ROHs (each had a single long ROH; Figure 2D; Figure S3).

#### 3.2.4 Comparison

Beyond intraspecific variation in genetic diversity and ROH, we also aimed to compare these metrics across species. Generally, SNP H_O_ was higher in brown bears (Figure 2A). Relative to the species examined, polar bear SNP H_O_ values were lower and fell within the range for American black bears. Genome‐wide H_O_ values contrasted more starkly, with no overlap between species; American black bears had the highest values, polar bears had the lowest value, and brown bears spanned between the two other species, displaying the greatest range of H_O_ (Figure S1). Across species, polar bears and admixed American black bear populations had the lowest F_ROH_ values. Low‐diversity American black bear populations have similar F_ROH_ to high‐diversity brown bear populations (Figure 2B).

The ROH burden also varied among species. The number of ROHs among black and brown bears overlaps, except for an outlier with a particularly high number of ROHs and total ROH lengths (i.e., the Apennine brown bear). Additionally, brown bears exhibit longer total lengths of ROHs than black bears. Polar bears exhibit a consistently low number of ROHs and short total lengths (Figure 2C,D).

### 3.3 Evaluation of Deleterious Variation among Species and within Species Lineages

**FIGURE 3:**
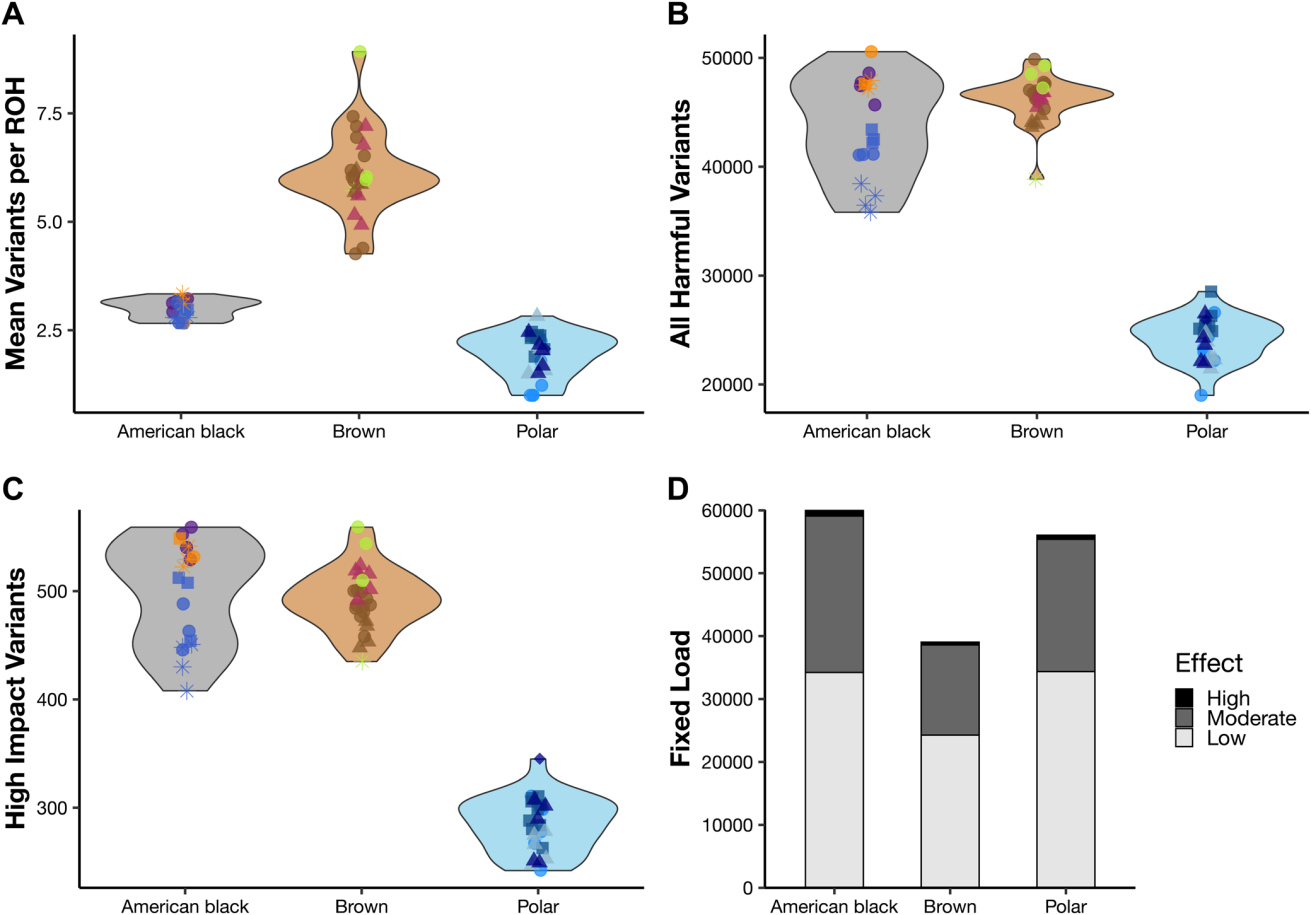
Distribution of harmful variants for each bear species ( *Ursus americanus* : Gray; *U. arctos* : Light brown; *U. maritimus* : Light blue; point symbology corresponds to Figure 1). The distribution of (A) all putatively harmful and (B) high‐impact/loss‐of‐function variants identified via SnpEff. (C) The mean number of deleterious variants per ROH tract across the individuals sampled. (D) Total fixed load for each focal species as stacked bars, with high‐effect variants in black, moderate‐effect variants in dark gray, and low‐effect variants in light gray.

**FIGURE 4:**
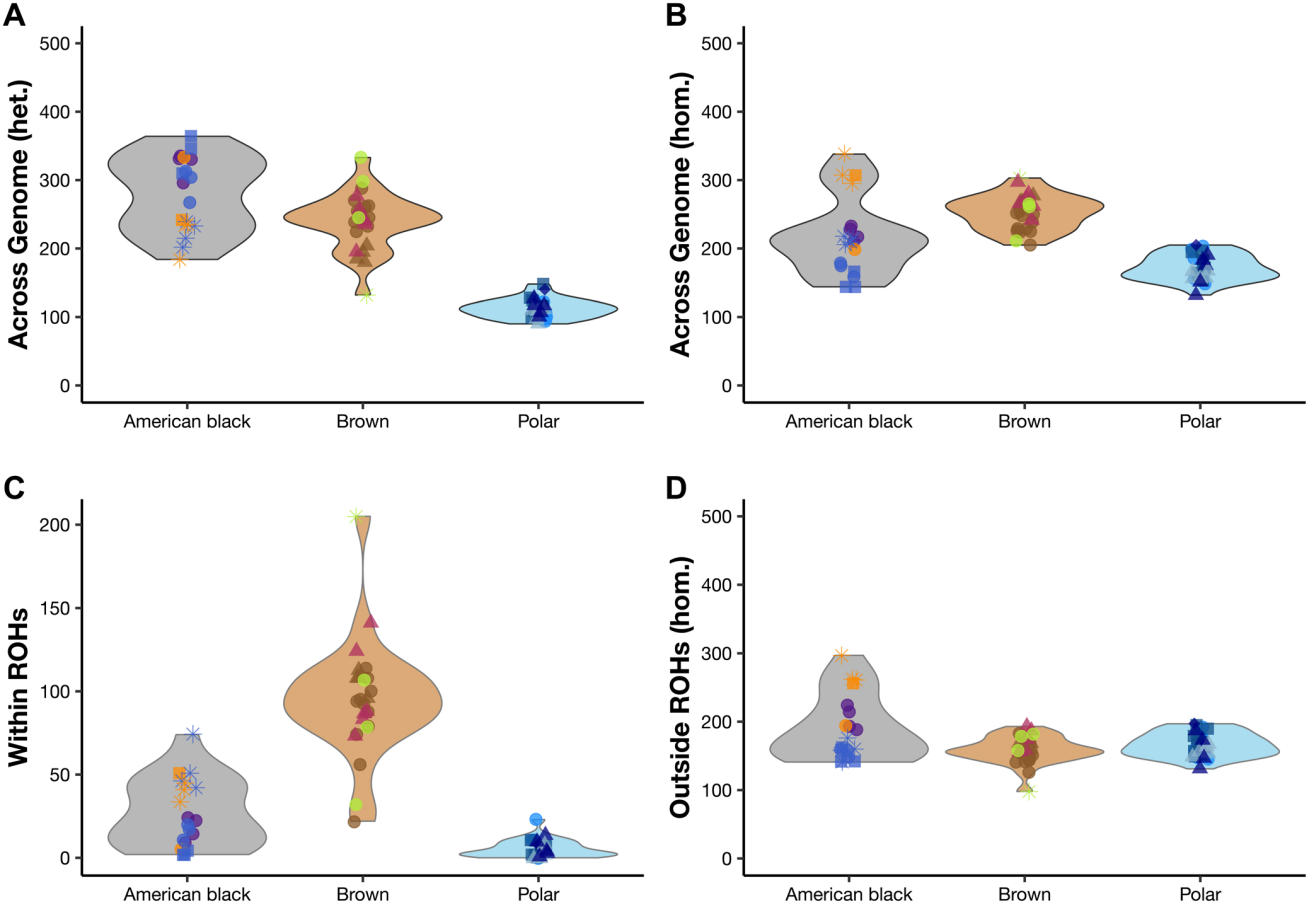
Distribution of high‐impact/loss‐of‐function harmful variants for each species ( *Ursus americanus* : Gray; *U. arctos* : Light brown; *U. maritimus* : Light blue; point symbology corresponds to Figure 1). Variants identified as (A) heterozygous (i.e., potential load) and (B) homozygous (i.e., realized load) within each individual. Homozygous data was further classified as being found (C) within or (D) outside of ROH tracts.

To understand genetic variation among these species, we estimated the total number of variants within the dataset and on a per‐individual basis, then estimated how much of that variation was deleterious. Within 19 American black bears, 17.8 M variants were identified, which ranged between 4.1 and 5.7 M per individual (mean = 5.0 M; SD = 0.5 M; Table S1). Black bears averaged 43.7 k (SD 4.5 k) deleterious variants per individual (Figure 3B), of which an average of 498 variants were estimated by SnpEff as “high impact,” meaning they had the greatest predicted effect on protein function (Figures 3C, 4; Table S1). We observed geographic patterns of deleterious variation, such that the eastern lineage had the lowest number of variants, and western lineage samples had a higher deleterious load. Notably, only 89% of the genome was investigated for black bears; thus, our results may reflect an underestimation of both total and deleterious variation compared to the other species. For brown bears, a total of 11.7 M variants were identified among 25 bears, ranging between 4.7 and 5.8 M per individual (mean = 5.4 M; SD = 0.2 M; Table S1). Brown bears averaged 46.2 k (std 2.1 k) deleterious variants per individual with minimal geographic variation among the lineages (Figure 3B). However, the Apennine bear had fewer deleterious variants (38.9 k) than other brown bears in this study. Of the total deleterious variants, an average of 492 were annotated as high impact per individual (Figure 3C). Among the 25 polar bear individuals, there were 5.0 M variants with 1.0–1.4 M within each animal (mean = 1.3 M; std. = 0.1 M; Table S1). The polar bear had 24.0 k (std 1.9 k) deleterious variants per individual, of which an average of 285 were highly deleterious (Figure 3B,C).

Total fixed load was highest among black bears (60,093 variants), followed by polar bears (56,040) and brown bears (39,044; Figure 3D). The fixed variants that were considered high effect made up a small proportion of these totals (1.7% for American black, 1.2% for polar, and 1.1% for brown bears). Most fixed variants fell into moderate or low effect categories, with the overwhelming majority being missense variants of moderate effect (24,872 for American black, 21,019 for polar, and 14,344 for brown bears) and synonymous variants of low effect (33,011 for American black, 33,705 for polar, and 23,836 for brown bears).

We also considered our variable load data from the perspective of potential and realized load by identifying which alleles were present within each individual, respectively, in a heterozygous or homozygous state. Broadly, polar bears had the lowest potential and realized load when low‐, medium‐, and high‐impact classes were considered together, but there was substantial overlap between brown and American black bears (Figure S4). The overall patterns among species were driven by the patterns for low impact deleterious alleles (Figure S5). Among moderate‐ and high‐impact alleles (Figure 4; Figure S6, respectively), black bears contained the highest potential load, followed by brown and then polar bears. Realized load was similar between polar bears and most black bear populations for the most impactful variants, though brown bears had higher realized load comprised of moderate‐ and high‐impact variants than all but a few American black bear populations (Figure 4B–D).

ROH tracks are known to harbor increased amounts of deleterious variation that have not had the same exposure to selection. Thus, we considered two metrics to associate deleterious variation with an ROH track. Among all three species of bears, more of the realized load occurred within rather than outside of an ROH track (Figure S4); however, this pattern was reversed for variants annotated to have the highest impact on protein function (Figure 4). Among both American black and brown bears, populations known to have gone through bottlenecks had the most high‐impact variants within ROH tracks compared to individuals across the range (Figure 4C). Secondarily, we estimated the number of deleterious variants (of any impact) per ROH track per individual. Brown bears averaged 6.12 deleterious variants per ROH (Figure 3A), whereas American black and polar bears averaged 3.00 and 1.94 variants per ROH. While the number of deleterious variants per track was higher in brown bears, the range of total harmful variants per individual was similar between brown and American black bears (Figure 3B).

### 3.4 Historical Effective Population Size, Genetic Diversity, Harmful Variation, and ROH Distribution

There was high similarity in the overall shape of individual PSMC’ estimates from samples across the range within species, as well as the magnitude of the historical N_E_ estimate (Figure S7). However, we observed that populations with known inbreeding show truncated PSMC’ curves, particularly at the oldest time segments.

**FIGURE 5:**
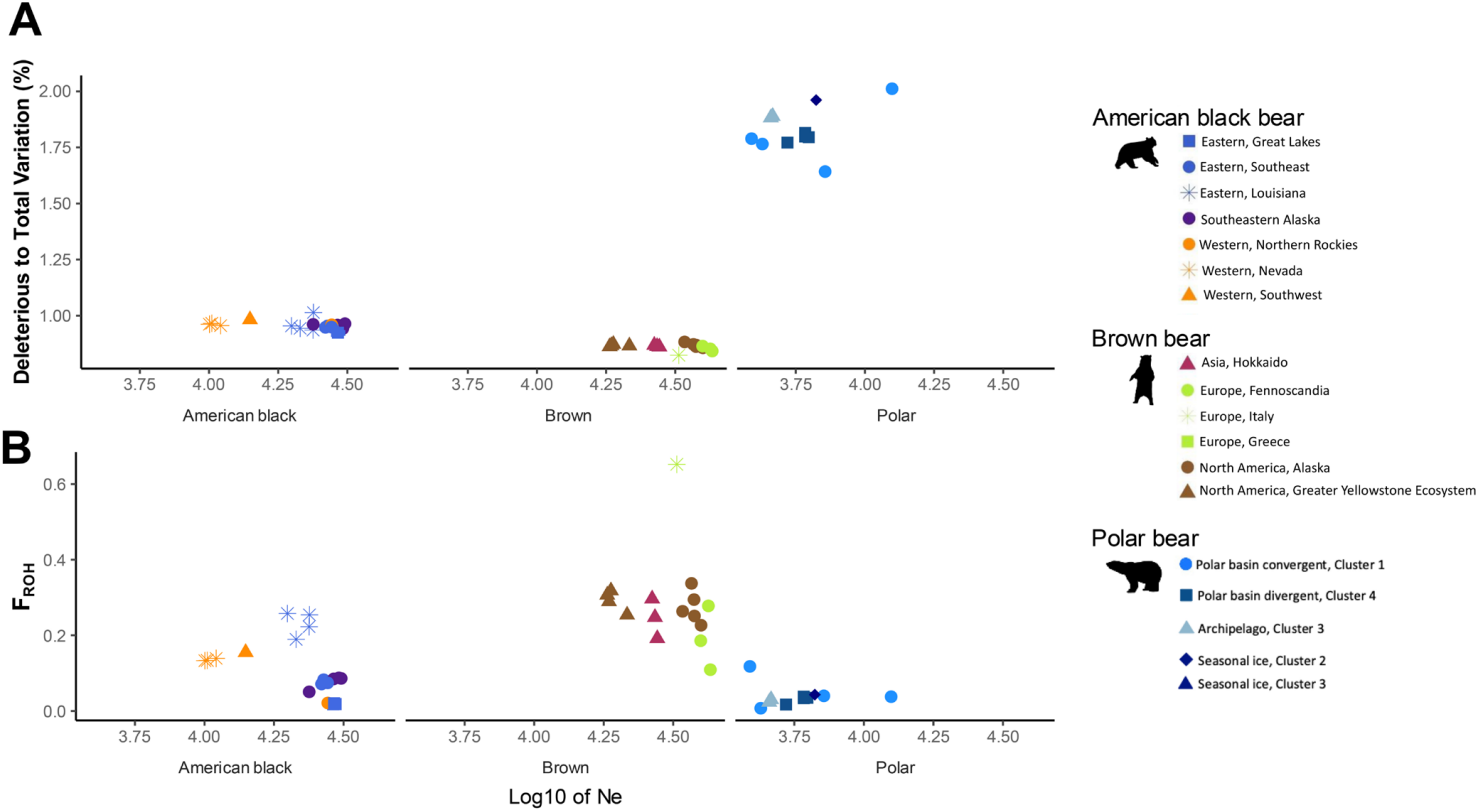
Comparison of genetic load and ROH burden to evolutionary history within three bear species: American black bear ( *Ursus americanus* ), brown bear ( *U. arctos* ), and polar bear ( *U. maritimus* ). (A) Proportion of deleterious variants out of total variants (in percent) in relation to the geometric mean of the effective population size (N_E_) for each individual (color and point symbology corresponds to Figure 1). The N_E_ values were ascertained using PSMC’ and log_10_‐transformed. (B) The proportion of the genome comprised of ROH tracts (F_ROH_) in relation to the geometric mean of N_E_ for each species.

Generally, there was a positive correlation between the geometric mean of the historic N_E_ and both the total number of genetic variants detected and the total number of putatively deleterious variants per individual (Figure S8A,B). This relationship reversed when looking at the proportion of putatively deleterious to total number of variants (Figure 5A), and the relationship was more pronounced when considering only the subset of homozygous deleterious variants (Figure S8C). These patterns held when looking within species but were less clear within polar bears than within the other ursids. The counts of variants (both total and deleterious) show different trajectories along the Eastern and Western lineages of the American black bear, with admixed Alaskan samples falling at the intersection of the two lineages (Figure S8A,B).

Relationships between the geometric mean of the historic N_E_ and H_O_ or F_ROH_ values were not expected to be as clear, since H_O_ and F_ROH_ can be strongly affected by recent demographic history. There was a generally positive correlation between N_E_ and both H_O_ and F_ROH_ when looking at individuals across all species (Figure 5B; Figure S8D). Within species, this trend was stronger with SNP H_O_ and between polar bears and brown bears genome‐wide but was less evident with the genome‐wide H_O_ estimates for American black bears (Figure S8D,E). Within species, F_ROH_ appeared to be more impacted by contemporary bottlenecks and inbreeding than historic population sizes.

## 4 Discussion

### 4.1 Demography and Load

Theory predicts that genetic diversity, including potentially harmful variation, will be greater in populations with larger historic N_E_ (Bertorelle et al. 2022). However, this pattern can be disrupted when demographic processes impact selection, resulting in either purging as harmful variants are exposed to selection in homozygous states or in an increased frequency of harmful alleles when drift is stronger than selection (Bertorelle et al. 2022; Bosse and van Loon 2022; Wilder et al. 2023). Populations with larger historic N_E_ have received particular scrutiny and concern recently, given that these populations implicitly carry a higher masked load; changes in their demographic trajectories can shift the ratios of neutral, beneficial, and harmful variants that comprise their overall diversity (Kyriazis et al. 2021).

Our hierarchical sampling scheme helped showcase the interplay between historic effective population size and contemporary demography in shaping genetic diversity and the distribution of genetic load. Looking across our samples, we saw that variation, both neutral and deleterious, increased alongside historic N_E_ (Figure S7A,B). However, load made up a lower proportion of total variation with increasing N_E_—especially realized load (Figure 5A; Figure S8C). Our results suggest that breaks in this pattern could signal genetic erosion. We saw that small, isolated American black bear populations and the highly inbred Apennine brown bear did not fit these trends as closely. Southwestern American black bears had the lowest historic N_E_ and relatively high total variants, while Louisiana bears (known to be of management concern) had higher historic N_E_ but lower total variants. The Apennine brown bear had a higher proportion of harmful variants than other brown bears of similar historic N_E_, indicating fixation of harmful variants. Additionally, while bias between species in the number of annotated variants is expected, the differences in total variants between polar bears and brown and black bears were pronounced and seemed to be driven by low historic N_E_ (Figure S8A). Interestingly, counts of variants in relation to historic N_E_ were correlated across American black bears but also exhibited different slopes between the Eastern and Western lineages (Figure S8A,B). All polar bear individuals clustered closely together with the lowest counts of variants and N_E_ estimates, while brown and black bear individuals overlapped with one another toward the upper ranges of both variant counts and N_E_ estimates.

In contrast, heterozygosity and F_ROH_ are more affected by contemporary processes than total variation, and deviation from trends in N_E_ could potentially be used as a diagnostic to identify populations that warrant management interventions. Most of the bear populations we examined exhibited correlations between historic N_E_ and both genome‐wide and SNP H_O_ (Figure S8D,E), as well as N_E_ and F_ROH_ (Figure 5B). The exceptions were populations known to be isolated (e.g., Apennine brown bears) or that have experienced contemporary population reductions (e.g., American black bears in Louisiana), which had lower H_O_ than expected for their historic N_E_, as well as American black bears in Nevada, which are recolonizing former habitat and had higher genome‐wide H_O_ than expected for their historic N_E_. These patterns may help discern between populations that are experiencing genetic erosion caused by contemporary demographic changes, as opposed to populations where long‐term evolutionary processes have generated lower diversity or higher genetic load without causing reductions in fitness.

It is typical to see genetic load masked in heterozygous states within large, interconnected groups and expressed in small, isolated groups (Mathur and DeWoody 2021). While there was a large amount of inter‐ and intraspecific variation in the relationship between potential and realized load across our study species, our data fit expectations. Most notably, the populations expected to be isolated, bottlenecked, and undergoing inbreeding showed the highest proportion of harmful variants falling within ROHs (i.e., Louisiana black bears and the Apennine brown bear).

Fixed load, i.e., harmful variants fixed within all individuals across a species, typically arise as a result of stochastic processes under low N_E_. As such, it was not surprising to see the lowest fixed load among brown bears, which have had the highest historic mean N_E_ (Figure 3D). In contrast, the fixed load among polar bears was notably higher than their total variable load. This pattern suggests that their long‐term low N_E_ resulted in both greater fixation of low and moderate effect variants, while also allowing genetic purging of high effect variants. The higher fixed load in polar bears may also be a consequence of genetic drift due to the strong selection on traits advantageous to a narrow environmental niche (Gillespie 2010). Selective sweeps during the polar bear's adaptation to the Arctic could have resulted in the fixation of deleterious variants via hitchhiking (Samaniego Castruita et al. 2020; Sun et al. 2024). Alternatively, many putatively deleterious variants as assigned via annotation software could be functionally adaptive in the context of an organism's environment. In effect, the fitness consequences of fixed load are generally of greater forgone than current significance and therefore difficult to target for management outside of synthetic biology; the presence of deleterious variants may affect the trajectory of future advantageous alleles that arise within a population.

### 4.2 Signatures of Inbreeding and Diversity

Certain historic demographic processes can produce a non‐linear relationship between the number of ROH tracts observed and the total lengths of these tracts. Many of the bear populations sampled exhibit a straightforward positive linear relationship between the number of ROHs and their sum lengths. However, polar bears and most American black bears show a pattern of few and short ROHs, which is associated with admixture. While this conforms to previous studies in black bears (Puckett et al. 2015; Puckett and Davis 2021), the pattern in polar bears may more accurately reflect the circumpolar genetic structure of this species (Kutschera et al. 2016; Malenfant et al. 2016; Peacock et al. 2015). Exceptions were found among American black bears in the southwest and Louisiana. These bears have more ROHs that are greater in length, which can be attributed to population bottlenecks. Additionally, the Asian and North American brown bears show a pattern of greater ROH length relative to the number of ROH tracts, which can suggest contemporary consanguineous matings (Ceballos et al. 2018). Across their range, brown bears exhibited ROHs of lengths greater than those found in the other species examined (Figure S3C), suggesting contemporary inbreeding may be widespread in this species.

Often the relationship between heterozygosity and F_ROH_ is oppositional: groups with high heterozygosity have low F_ROH_, and groups with low heterozygosity have high F_ROH_. Neither brown nor polar bears fit this trend at the species level. Brown bear individuals had both the highest SNP H_O_, moderate genome‐wide H_O_, and the highest F_ROH_, whereas polar bears had the lowest H_O_ and F_ROH_ (Figure 2A,B; Figure S1). Similarly, relative values between lineages and regions did not adhere to this expectation; i.e., the highest and lowest values between groups did not simply flip between F_ROH_ and H_O_. However, with few exceptions, the expected oppositional pattern was observed at the individual level.

While genetic diversity is an important component of species resilience, comparing heterozygosity between species as a proxy measure of diversity highlighted that it does not provide a complete measure of genetic health (DeWoody et al. 2021). For instance, while polar bears had the lowest overall heterozygosity, their largest threat is loss of sea ice—not genetic erosion. Additionally, while American black bears had either comparable SNP H_O_ or higher genome‐wide H_O_ than brown bears, this did not bar American black bears from having highly isolated populations of management concern (Figure S1; Murphy et al. 2018). Outlier values among species may be more useful indicators than absolute values, as we found outliers corresponded to known bottlenecked populations.

### 4.3 Species‐Level Insights

American black bears are forest generalists and ecological opportunists that show little obvious evidence of local adaptation. Genetic exchange has occurred across much of their range prior to contemporary conditions (Puckett et al. 2015). Land‐use change and predator persecution have led to bottlenecks and persisting isolation, largely among the southernmost portion of their range (Garshelis et al. 2016). The range of heterozygosity values and distribution of ROH burden suggest that, while most American black bears warrant their ‘least concern’ designation, inbreeding is resulting in realized genetic load in some populations that have experienced recent bottlenecks. This is further underscored by differences in long‐term N_E_ between southwestern and Louisiana bears. Louisiana bears had higher historic N_E_ than southwestern bears but higher F_ROH_ and a higher proportion of harmful to total variation, suggesting that contemporary bottlenecks may be resulting in stronger inbreeding depression within this population (Murphy et al. 2018). In contrast, Arizona bears fall within a larger southwestern group with structure dictated more by Pleistocene–Holocene vicariance than post‐colonial changes, and Nevada bears are recolonizing former habitat without showing signals of bottlenecking (Gould et al. 2022; Lackey et al. 2013; Malaney et al. 2018). The likely inbreeding depression among Louisiana bears may warrant management interventions, including facilitated translocations and monitoring of genetic health.

Brown bears have the largest geographic range among bears and have adapted to incredibly varied habitats, which may account for the high levels of genetic diversity detected across their populations. Heterogeneous selective pressures, sufficient population size, and population subdivision from regional isolation have produced a wide array of local adaptations across these bears, from arctic to desert environments (Benazzo et al. 2017; de Jong et al. 2023; Tumendemberel et al. 2023). They also have not experienced the same degree of historic bottlenecks as found in polar and American black bears (Armstrong et al. 2022). However, relatively recent bottlenecks and ongoing recolonization following incomplete range contraction may explain the nearly ubiquitous presence of long ROHs indicative of contemporary inbreeding (Figure S3C, Karamanlidis et al. 2018; Swenson et al. 2011). These results are in concordance with other recent work in brown bears showing high F_ROH_ composed of many long ROHs across the species' range (de Jong et al. 2023). Given that deleterious variation increases commensurately with overall diversity, it follows that realized load would increase within a species with high diversity as populations undergo conditions that increase homozygosity. This may be or become an issue of management concern within more vulnerable brown bear populations. Serial founder effects in expanding populations may also foster gene surfing and drift even among growing populations (Hallatschek et al. 2007; Tensen et al. 2024). However, with the exception of Apennine bears, most metrics of diversity and load across brown bears were fairly consistent across populations, even with variation in their historic N_E_. Careful management will likely be necessary for the persistence of the Apennine bears, along with bears in Pakistan and the Gobi Desert, which exhibit similarly low genetic diversity (Tumendemberel et al. 2019, 2023). In other populations, sustained demographic recovery may permit some brown bear populations to purge their genetic load as it becomes exposed to selection.

Polar bears are highly specialized to arctic marine environments and have persisted at population sizes where deleterious variation appears to have been subject to strong selection, resulting in genetic purging (Benazzo et al. 2017; Liu et al. 2014). We identified a lower genetic load and burden of ROHs. However, the proportion of total identified variants annotated as putatively harmful—and expressed in homozygous states—was highest within this group and followed a trend of negative correlation with the historic effective population size (Figure 5; Figure S7C). Given the alarming rates of environmental change occurring in the polar regions of the world due to increasing temperatures, genetic load may be less of a problem than limited evolutionary potential in this species. Polar bears may also be considered at risk of mutational drought—i.e., falling below a sufficient population size required to generate new, beneficial mutations (Mawass et al. 2024). Genetic forecasting suggests climate maladaptation is likely to occur among polar bears in the Canadian high Arctic, though other groups of polar bears are expected to be similarly at risk (Rivkin et al. 2024).

### 4.4 Limitations and Caveats

One of the promises of analyzing ROH length distributions is the ability to time demographic events in more recent generations (Humble et al. 2023; Kirin et al. 2010). ROH lengths are inversely correlated with generations elapsed since inbreeding, since recombination events break up ROH tracts. However, our experience with comparisons between species has highlighted some concerns with such interpretations of ROH distributions. First, there is no set standard for the methods used to assign ROH tracts to size classes associated with time since inbreeding. Some studies employ uniform and arguably arbitrary cut‐offs across species, while others cluster based on tract lengths within the group analyzed (Benazzo et al. 2017; Foote et al. 2021; Marsden et al. 2016; Pemberton et al. 2012; Saremi et al. 2019). Second, detection of ROHs of particular tract lengths can also vary due to characteristics of the assembly used or the parameters employed in detection algorithms. For instance, the detection of long ROHs may be reduced when they extend beyond the length of shorter scaffolds (which can also result in underestimation of total F_ROH_). In our data, detection of long ROHs may be reduced among brown and polar bears relative to black bears due to differences in scaffold lengths. However, we found that brown bears had a notably larger distribution of longer ROHs compared to the other bear species, to the extent that clustering ROHs into size classes across the three species did not lend to straightforward interpretation of the timescales associated with ROH lengths. Recently published ROH analyses of brown bears using assemblies with longer scaffolds found even greater numbers of these very long ROHs (de Jong et al. 2023; Lindahl 2023). While this should not affect intraspecific comparisons, the effects of differing scaffold lengths in assemblies could reduce certainty in interspecific comparisons and may obfuscate attempts to interpret demographic histories from ROH length data since the detected tract lengths are not solely determined by recombination events. Detection methods can also produce varied results; for instance, the stricter definition of heterozygosity in sliding windows applied in our PLINK parameters produced lower F_ROH_ estimates for polar bears compared to Benazzo et al. (2017), where estimates of diversity were weighted by genomic tract length. Taken as a whole, the variability in measures between species due to true biological difference and within‐species genomic resources and software parameters suggests a need for caution and thoughtful consideration of methods and interpretation (Silva et al. 2024).

We acknowledge that we used samples of varying depths (14—55×; Table S1) in this work, which would have the greatest impact on heterozygous calls. The expected effect of missing heterozygous sites would be an underestimation of heterozygosity, potential load, and historic N_E_, and an overestimation of F_ROH_ and realized load. A series of post hoc two‐sample t‐tests found no significant differences in our measures of H_O_ or ROH burden between samples with depth of coverage below and above 20×, as well as no significant differences in the total number of variants detected and the number of deleterious variants.

### 4.5 Choosing between Data Depth and Sampling Breadth in Conservation Genomics

Efforts to conserve and manage wild populations must balance trade‐offs between the costs of data and the value of their utility. Previous studies using individual representatives of species have been able to identify trends in diversity, load, and ROH pertaining to demographic history and trophic level, body size, and latitude (Brüniche‐Olsen et al. 2018; Zoonomia consortium 2020). However, sampling error caused by random sampling of individuals from species with population‐level variation can result in biased representation of the species as a whole.

In our data, while the distribution of diversity, load, and ROH was consistent across polar bears, greater variation was observed between individual brown bears and notably between populations *within* lineages of American black bears. For instance, a random individual black bear sampled from coastal Louisiana would be likely to have many long ROHs, a high F_ROH_, a high realized load, and low overall diversity. These values would be nearly inverse if a random individual were to be sampled from the Great Lakes region, where populations are admixed. As such, individuals do not make good representatives of species distributed across wide ranges with varied habitats and diverse demographic contexts, such as black and brown bears.

Prior knowledge of species can help inform whether there is greater benefit to sampling more individuals at lower depth or fewer individuals at greater depth to obtain the metrics discussed here. Particularly, the amount of population structure and the extent of phenotypically observable local adaptation ought to inform these decisions, as evolution takes place at the population level. In species with wide ranges and varied demographic histories among populations, trends warranting management intervention may be more clearly revealed by sampling more individuals and foregoing greater depth. The insights of such a study design will be further facilitated with the development or refinement of methods for ROH and PSMC estimation from low‐depth whole‐genome resequencing. In contrast, sampling fewer individuals with greater depth and resolution may be a preferred trade‐off in species such as polar bears, i.e., adapted to a specific environment and lacking strong genetic structure. In species with similar characteristics, the genomic signatures detected in one individual are more likely to encompass patterns seen more broadly within its species or ecoregion, and greater depth of coverage may allow for more certainty in analyses such as PSMC (Kutschera et al. 2016).

## 5 Conclusions

The evolutionary histories, contemporary demography, and environmental context of populations help in evaluating the degree of concern warranted and guiding strategies for interventions. In black bears, the composite of the metrics we gathered revealed a trend among bears in the Louisiana population that may be diagnostic for management intervention based on contemporary demographic changes. In brown bears, the Apennine bear consistently fell outside of the range of values in other populations, reinforcing what has been previously described about their history of isolation, inbreeding, and purging in this population. In polar bears, we did not observe regional trends in outlier values that warranted specific concerns for those populations with respect to genetic erosion.

Assessing load and inbreeding depression in the context of historic and contemporary demography can guide how and when management interventions should occur. For instance, genetic rescue may be performed between more closely associated populations if local adaptations are known to be present, such as with brown bears. Alternatively, individuals selected for assisted breeding programs or facilitated translocations may be subjected to a finer scale of individual scrutiny if there are high‐impact deleterious variants associated with extirpation risk (e.g., traits associated with reproduction or disease susceptibility). Recent work in giant pandas investigating these metrics was able to identify populations that should be targeted for future conservation efforts (Lan et al. 2024). Additionally, genetic interventions may be avoided altogether if it is determined that augmentation of suitable habitat alone is sufficient to maintain healthy populations over the long term. Genomic data, particularly when paired with simulation tools, can help determine whether interventions are necessary and which strategies are most likely to garner success (Kyriazis et al. 2023). Contrasting genomic signatures generated by long‐term evolutionary processes to those generated by contemporary demographic changes can help differentiate between populations more or less likely to persist with low diversity or high genetic load. Further, these measures can be tracked over time to monitor recovery success or emerging risks within managed groups.

## Conflicts of Interest

The authors declare no conflicts of interest.

## Supporting information


Figure S1.

Table S1.

## Data Availability

Whole genome sequences have been deposited in the NCBI SRA under BioProject PRJNA867575.
