# Supplementary material for: Linking Measures of Inbreeding and Genetic Load to Demographic Histories Across Three Species of Bears
Source: Evol Appl. 2025 Jul 16;18(7):e70133. doi: 10.1111/eva.70133 (PMC12267111; doi:10.1111/eva.70133)
Supplement: Supplementary file 1 — Figure S1. [file EVA-18-e70133-s002.docx]

**SUPPLEMENTAL FIGURES**


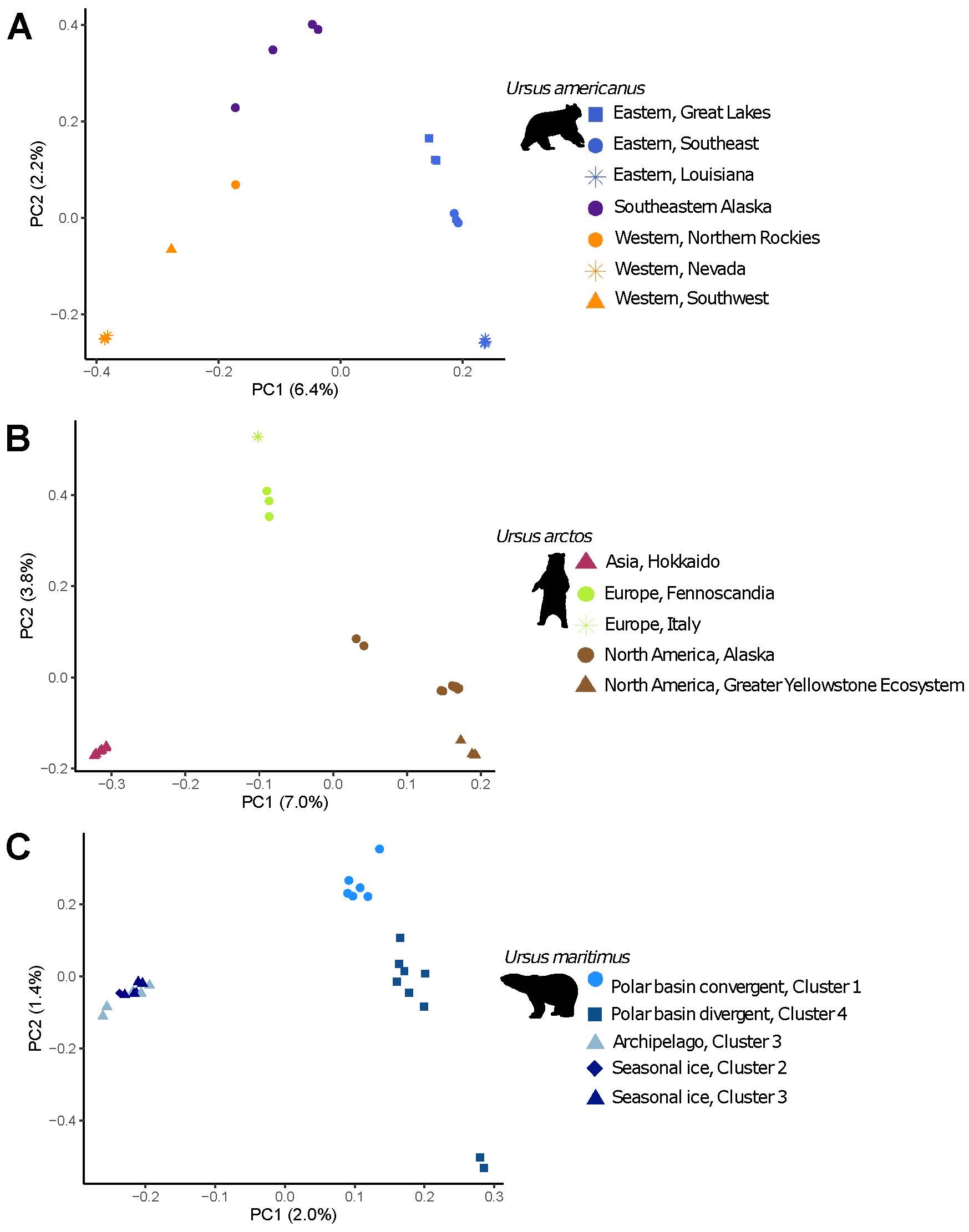


**Figure S1:** Principal components analysis of (A) *Ursus americanus* (American black bear)*,* (B) *U. arctos* (brown bear), and (C) *U. maritimus* (polar bear) samples. Lineages defined in comprehensive phylogeographic analyses are shown as unique colors within a species, where populations and/or geographic concordance is shown with diverse symbols.
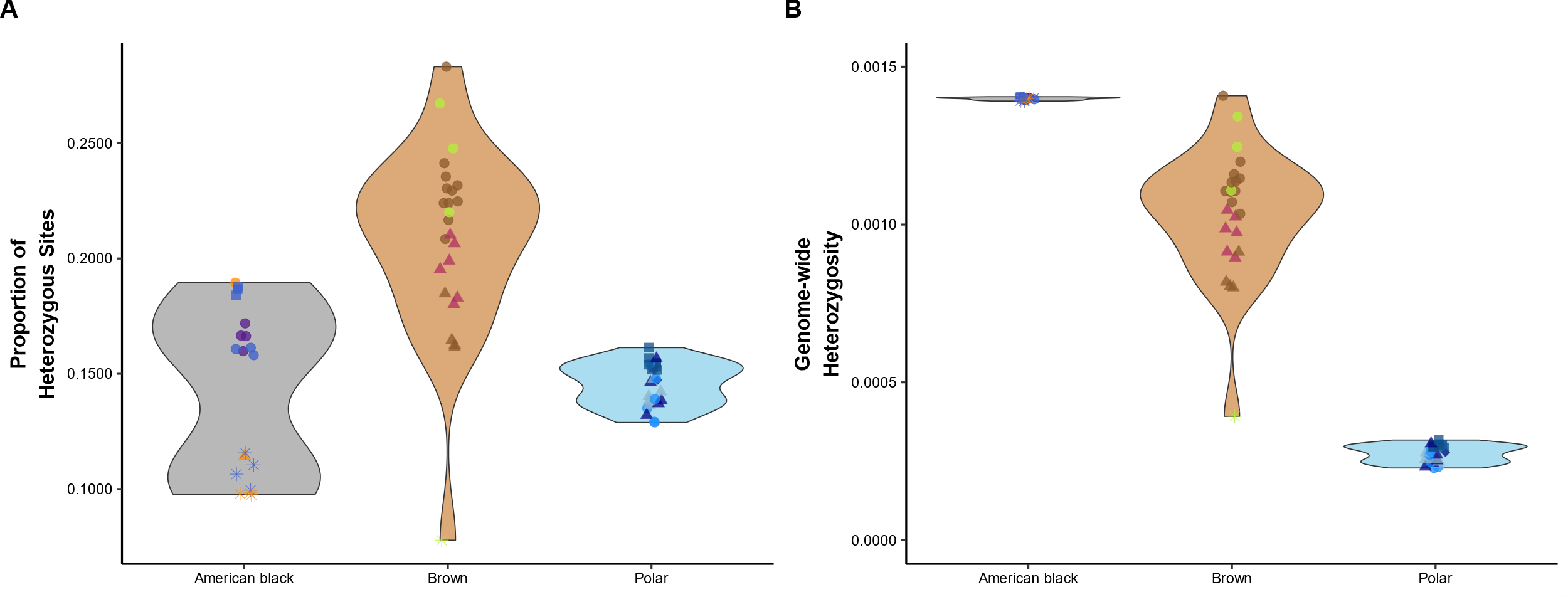


**Figure S2:** Heterozygosity estimates for individuals across each species. American black bears (*Ursus americanus*) are in gray, brown bears (*U. arctos*) are in light brown, and polar bears (*U. maritimus*) are in light blue, where point symbology corresponds to Figure S1. Heterozygosity is presented as both (A) SNP H_O_ (heterozygosity among all of the variable sites within the species dataset) and (B) genome-wide H_O_ (heterozygosity given the sum of the contig lengths of the reference assemblies).


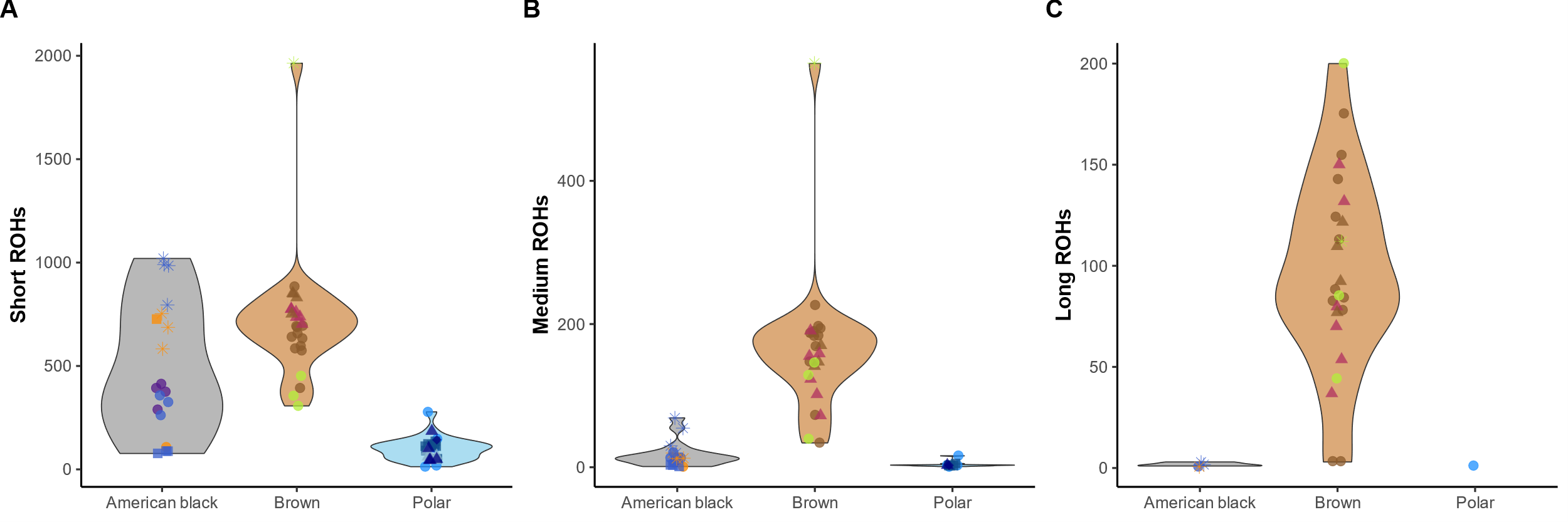


**Figure S3:** Counts of ROHs within individuals based on size classes. American black bears (*Ursus americanus*) are in gray, brown bears (*U. arctos*) are in light brown, and polar bears (*U. maritimus*) are in light blue, where point symbology corresponds to Figure S1. The number of (A) “Short” (tracts between 100-400kb); (B) “Medium” (tracts between 400-800kb); and (C) “Long” (tracts >800kb) ROH by species.


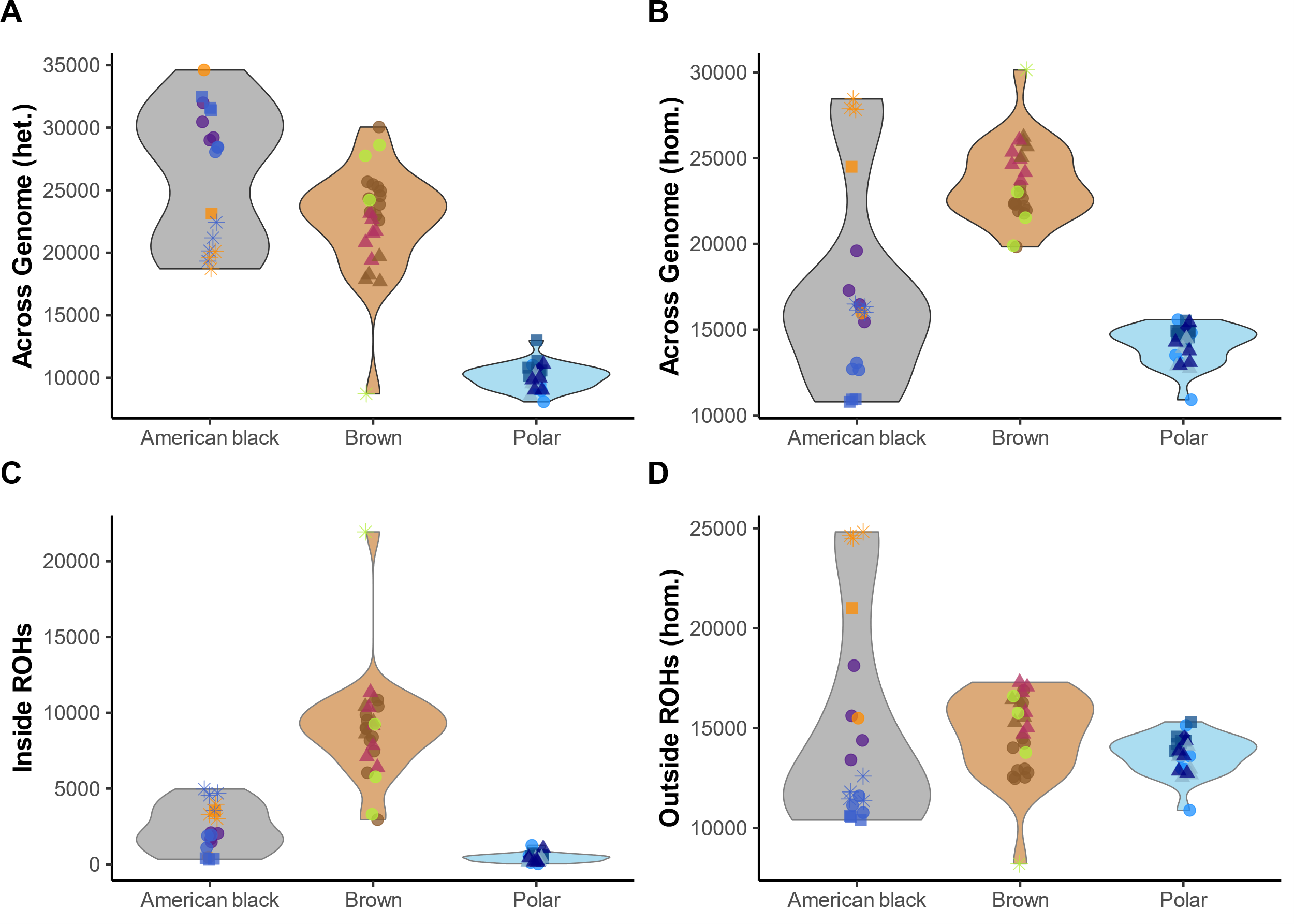


**Figure S4:** Counts of all identified harmful variants for each species. American black bears (*Ursus americanus*) are in gray, brown bears (*U. arctos*) are in light brown, and polar bears (*U. maritimus*) are in light blue, where point symbology corresponds to Figure S1. Putatively harmful variants in (A) heterozygous states (i.e., potential load) and (B) homozygous states (i.e., realized load). Counts of putatively harmful and homozygous variants located (C) within and (D) outside of ROH.

**
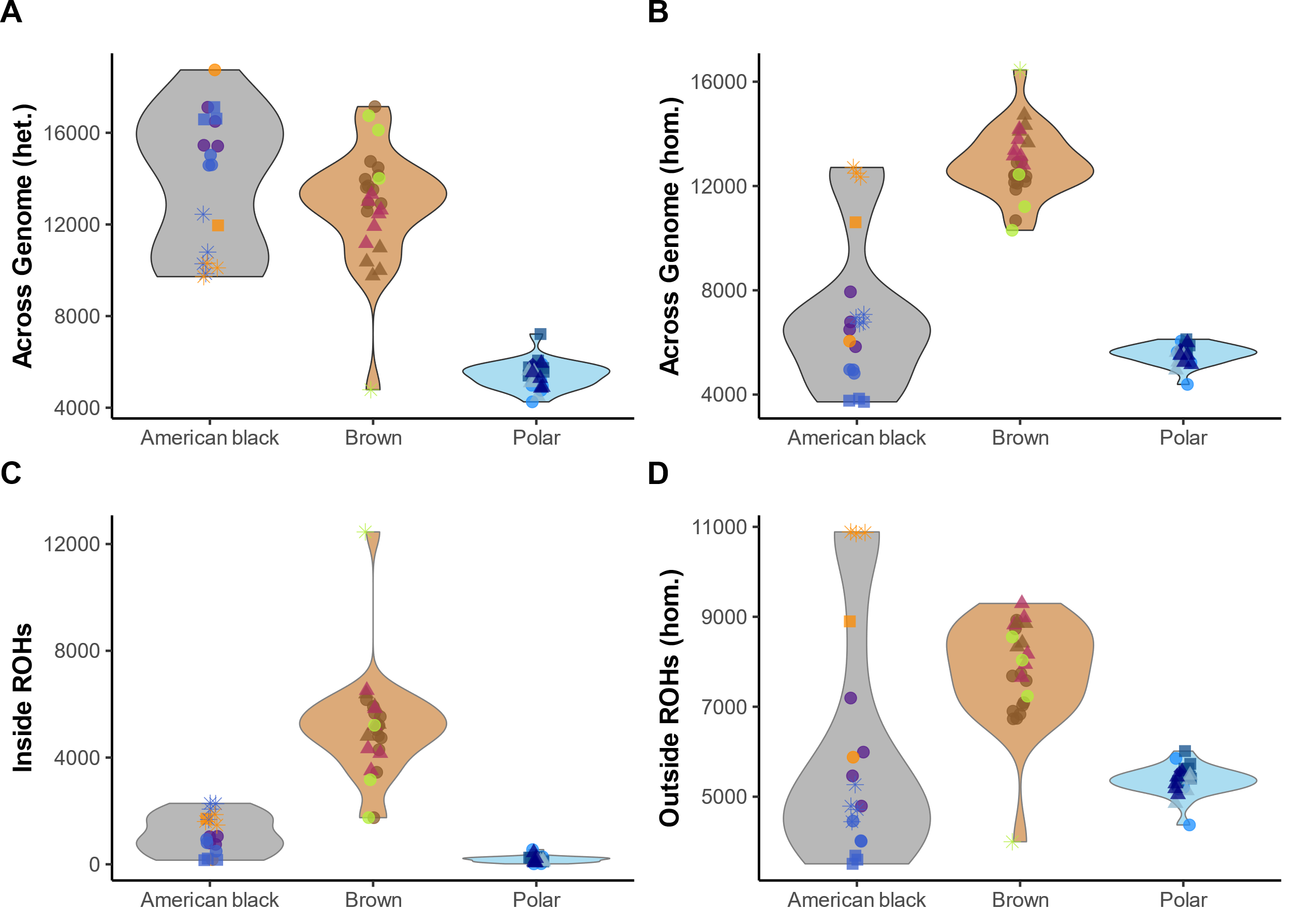
**

**Figure S5:** Counts of low-impact harmful variants for each species. American black bears (*Ursus americanus*) are in gray, brown bears (*U. arctos*) are in light brown, and polar bears (*U. maritimus*) are in light blue, where point symbology corresponds to Figure S1. Low-impact variants in (A) heterozygous states (i.e., potential load) and (B) homozygous states (i.e., realized load). Counts of putatively harmful and homozygous variants located (C) within and (D) outside of ROH.


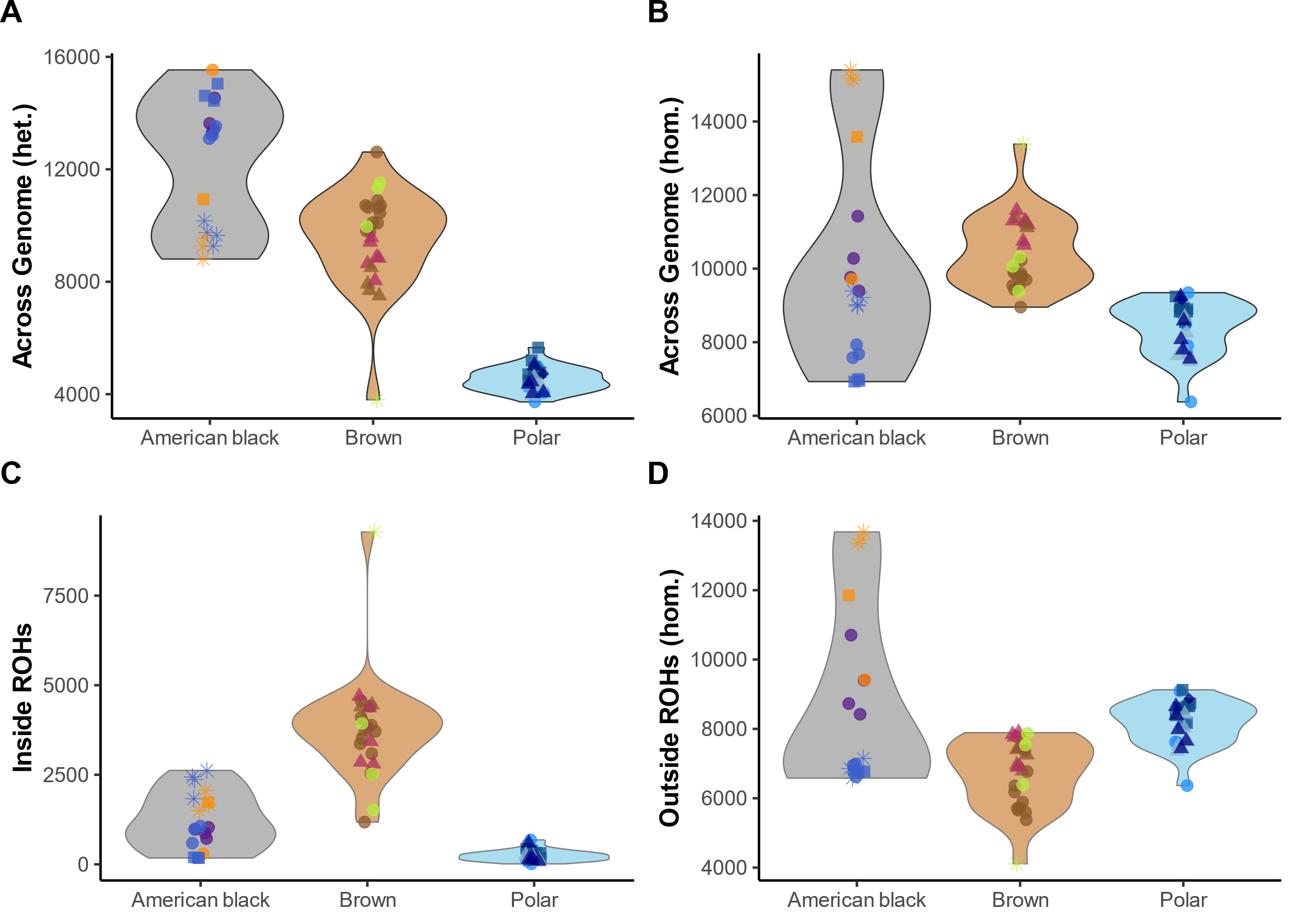


**Figure S6:** Counts of moderate-impact harmful variants for each species. American black bears (*Ursus americanus*) are in gray, brown bears (*U. arctos*) are in light brown, and polar bears (*U. maritimus*) are in light blue, where point symbology corresponds to Figure S1. (A) Moderate-impact variants in (A) heterozygous states (i.e., potential load) and (B) homozygous states (i.e., realized load). Counts of putatively harmful and homozygous variants located (C) within and (D) outside of ROH.


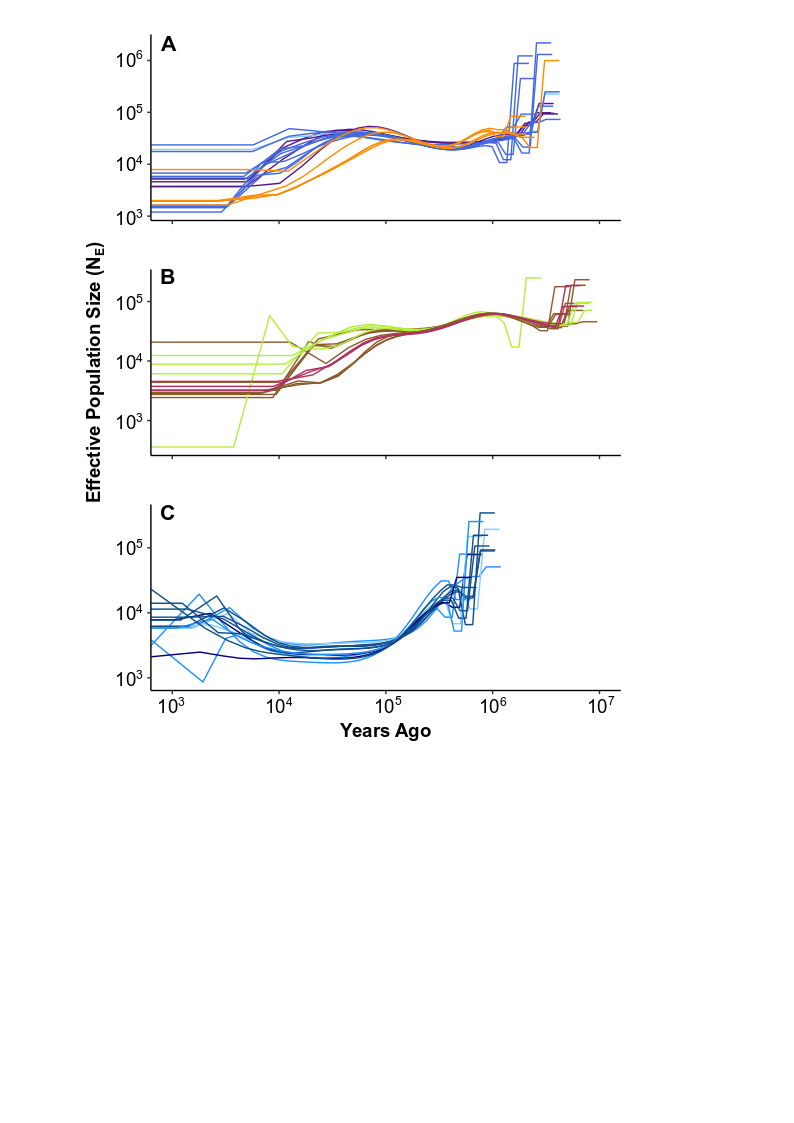


**Figure S7:** Plot of the change in effective population size (N_E_) over time, estimated for individual samples (i.e., two haplotypes; PSMC’) from MSMC2 for (A) American black bear (*Ursus americanus)*, (B) brown bear (*U. arctos)*, and (C) polar bear (*U. maritimus)*. Generations were scaled to years by using a time of 6.5 years per generation for American black bears (Hellgren and Vaughan 1989), and 10 years per generation for brown and polar bears (Skrbinsek et al. 2012). Colors represent the within species lineage for which the animal belongs. For American black bears: eastern lineage- blue, western lineage- orange, and Alaska- purple. For brown bears: Europe- lime green, Japan- maroon, and North America- brown. For polar bears: Archipelago- light blue, Seasonal Ice/Hudson Bay- navy, Polar Basin convergent- medium blue, and Polar Basin divergent- cerulean (also see Figures 1 and S1).


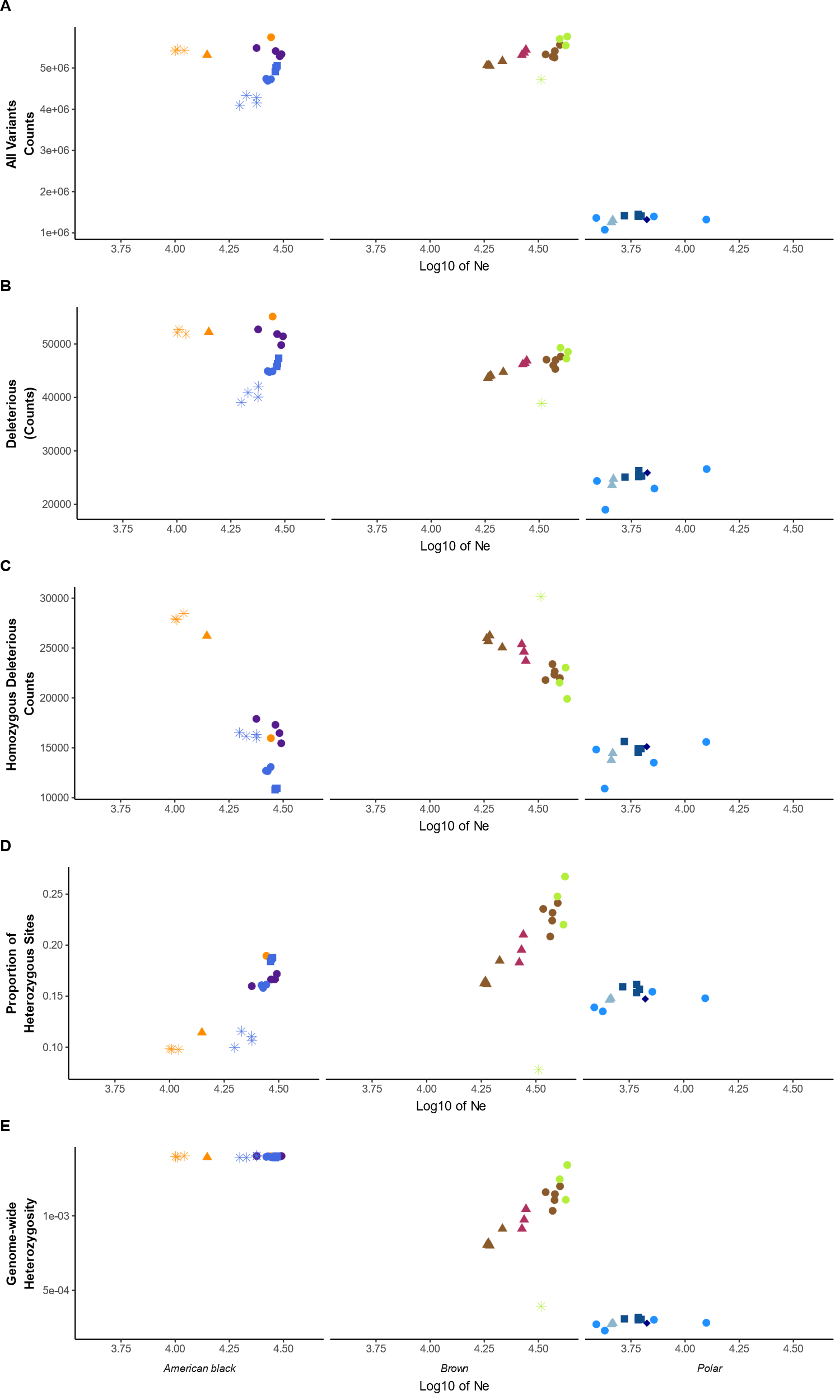


**Figure S8:** Distribution of counts of variants and heterozygosity in relation to the geometric mean of the effective population size (N_E_) for each species (*Ursus americanu*s, *U. arctos*, and *U. maritimus*; color and point symbology corresponds to Figure S1). Comparisons to historical N_E_ include (A) the total number of variants annotated by SnpEff; (B) the subset of putatively harmful variants; (C) realized load; (D) SNP H_O_; and (E) genome-wide H_O_. All estimates of N_E_ were based on PSMC’ and log10 transformed.

**REFERENCES**

Hellgren, E. C., & Vaughan, M. R. (1989). Demographic analysis of a black bear population in the Great Dismal Swamp. *The Journal of wildlife management*, 54(3). 969-977.

Skrbinšek, T., Jelenčič, M., Waits, L., Kos, I., Jerina, K., & Trontelj, P. (2012). Monitoring the effective population size of a brown bear (Ursus arctos) population using new single‐sample approaches. *Molecular Ecology*, 21(4), 862-875.
